# Comprehensive assessment of heavy metal(loid) contamination and health risks in agricultural soils near the Menzel Bourguiba steel smelter, Tunisia

**DOI:** 10.1038/s41598-026-45034-x

**Published:** 2026-04-02

**Authors:** Abdelwaheb Aydi, Sinda Sifi, Sabrine Zaghdoudi, Viktoria Mikita, Péter Szűcs, Mohamed Hamdy Eid

**Affiliations:** 1 Department of Earth Sciences, Faculty Science of Bizerte, University of Carthage, Jarzouna, 7021 Bizerte, Tunisia; 2 Faculty of Earth Science, Institute of Environmental Management, University of Miskolc, Miskolc- Egyetemváros, 3515 Hungary; 3 Geology Department, Faculty of Science, Beni-Suef University, Beni-Suef, 65211 Egypt

**Keywords:** Heavy metals, Health risk assessment, Monte Carlo simulation, Sensitivity analysis, Steel smelter, Soil contamination, Environmental health, Diseases, Environmental sciences, Risk factors

## Abstract

This study assessed heavy metal(loid) pollution (As, Pb, Zn, Fe, Cd, Cu, Cr, Ni) and the associated health risks in agricultural soils near the Menzel Bourguiba steel smelter in northeastern Tunisia. The extent of contamination was quantified using multiple pollution indices, including the geo-accumulation index (Igeo) and enrichment factor. Health risks were evaluated using both deterministic and probabilistic approaches, incorporating Monte Carlo simulation to characterize uncertainty and Sobol sensitivity analysis to identify key influencing parameters. Mean heavy metal(loid) concentrations (mg kg^−1^) decreased in the order of Fe (28,136) > Zn (480.73) > Cr (184.85) > Pb (147.12) > Ni (73.5) > Cu (55.50) > As (15.97) > Cd (5.27). Pollution indices consistently identified Cadmium (Cd) and Lead (Pb) as the primary contributors to soil contamination. Enrichment factor analysis revealed extreme enrichment for Cd, with concentrations exceeding background levels by up to 58 times. Probabilistic risk assessment indicated a high potential for adverse health effects. For children, the non-carcinogenic Hazard Index for lead exceeded the safe limit of 1 in over 50% of simulations. The Total Carcinogenic Risk (TCR) for children surpassed the acceptable threshold (1 × 10^−4^) in over 80% of simulations for As, Cr, and Cd. For adults, the TCR for Cd exceeded this threshold in over 70% of cases. The 95th percentile of the simulated TCR for children reached 5.7 × 10^−3^, a value 57 times higher than the acceptable limit. Sobol analysis identified the soil ingestion rate as the primary source of uncertainty in the risk models (contributing 68–74%). These findings indicate that the soil contamination poses a significant health risk to the local population, warranting targeted intervention strategies.

**Supplementary Information:**

The online version contains supplementary material available at 10.1038/s41598-026-45034-x.

## Introduction

Industrial contamination of agricultural soils represents a critical environmental health challenge globally. Heavy metal pollution from metallurgical operations poses particularly severe risks to surrounding communities through multiple exposure pathways^1–5^. Steel smelting facilities are major sources of heavy metal emissions, releasing toxic elements including lead (Pb), cadmium (Cd), chromium (Cr), and arsenic (As) into the environment through atmospheric deposition, wastewater discharge, and solid waste disposal^6–10^.

The Mediterranean region faces increasing pressure from heavy metal contamination due to intensive agricultural activities and industrial development^11^. Tunisia exemplifies these challenges, as a developing country with significant metallurgical industries where agricultural lands surrounding industrial facilities serve dual purposes of food production and residential activities^12^. The Menzel Bourguiba steel smelter, established in 1962 and located in the Bizerte governorate, represents a significant point source of contamination in northeastern Tunisia. Historical operations have resulted in the uncontrolled release of pollutants including exhaust gases, domestic sewage, and industrial wastewater, raising concerns about environmental contamination and associated health risks for the surrounding agricultural community.

Heavy metal contamination in agricultural soils poses multifaceted health risks through various exposure pathways, including direct soil ingestion, inhalation of contaminated dust particles, and dermal contact during agricultural and recreational activities^13–15^. Children are particularly vulnerable due to their higher soil ingestion rates, lower body weights, developing organ systems, and increased time spent in contact with contaminated environments^16^. The health effects of heavy metal exposure encompass both non-carcinogenic effects (developmental delays, neurological impairment, organ dysfunction) and carcinogenic effects (increased cancer risk), depending on the specific metals and exposure levels^17–20^.

Traditional deterministic risk assessment approaches, while valuable, often fail to adequately characterize the uncertainty and variability inherent in environmental exposure scenarios. Probabilistic risk assessment methods, particularly Monte Carlo simulation, provide more robust and realistic risk characterization by incorporating parameter uncertainty and spatial variability^21–24^. Furthermore, global sensitivity analysis techniques such as Sobol indices enable identification of key parameters driving uncertainty in risk estimates, facilitating targeted data collection and intervention strategies^25^. The integration of multiple geochemical pollution indices including the geo-accumulation index (Igeo), enrichment factor (EF), contamination factor (CF), and pollution load index (PLI) with probabilistic health risk assessment provides a comprehensive framework for evaluating soil contamination and associated health risks^26,27^.

Despite the potential for significant health impacts, comprehensive probabilistic health risk assessments of heavy metal contamination around Tunisian industrial facilities remain limited. Previous studies have primarily employed deterministic approaches with limited uncertainty characterization, potentially underestimating or mischaracterizing actual health risks^19,28–30^. This study addresses this gap by integrating advanced probabilistic methods with comprehensive sensitivity analysis and multiple geochemical indices to provide robust scientific evidence for public health decision-making.

The study hypothesizes that long-term, uninterrupted industrial operations at the Menzel Bourguiba steel smelter have resulted in significant heavy metal(loid) contamination of surrounding agricultural soils, with contamination levels and associated health risks that exceed regulatory thresholds and warrant targeted intervention strategies.

This study aims to conduct a comprehensive health risk assessment of heavy metal contamination in agricultural soils surrounding the Menzel Bourguiba steel smelter using advanced probabilistic methods. Specific objectives include: (1) quantifying heavy metal(loid) concentrations in agricultural soils around the facility and determining the degree of contamination using multiple geochemical indices; (2) evaluating non-carcinogenic and carcinogenic health risks for children and adults using both deterministic and probabilistic approaches; (3) characterizing uncertainty and variability in risk estimates through Monte Carlo simulation; (4) identifying key parameters influencing risk estimates through global sensitivity analysis (Sobol indices); and (5) providing evidence-based recommendations for risk management and public health protection.

## Materials and methods

### Study area and site description

**Fig. 1:**
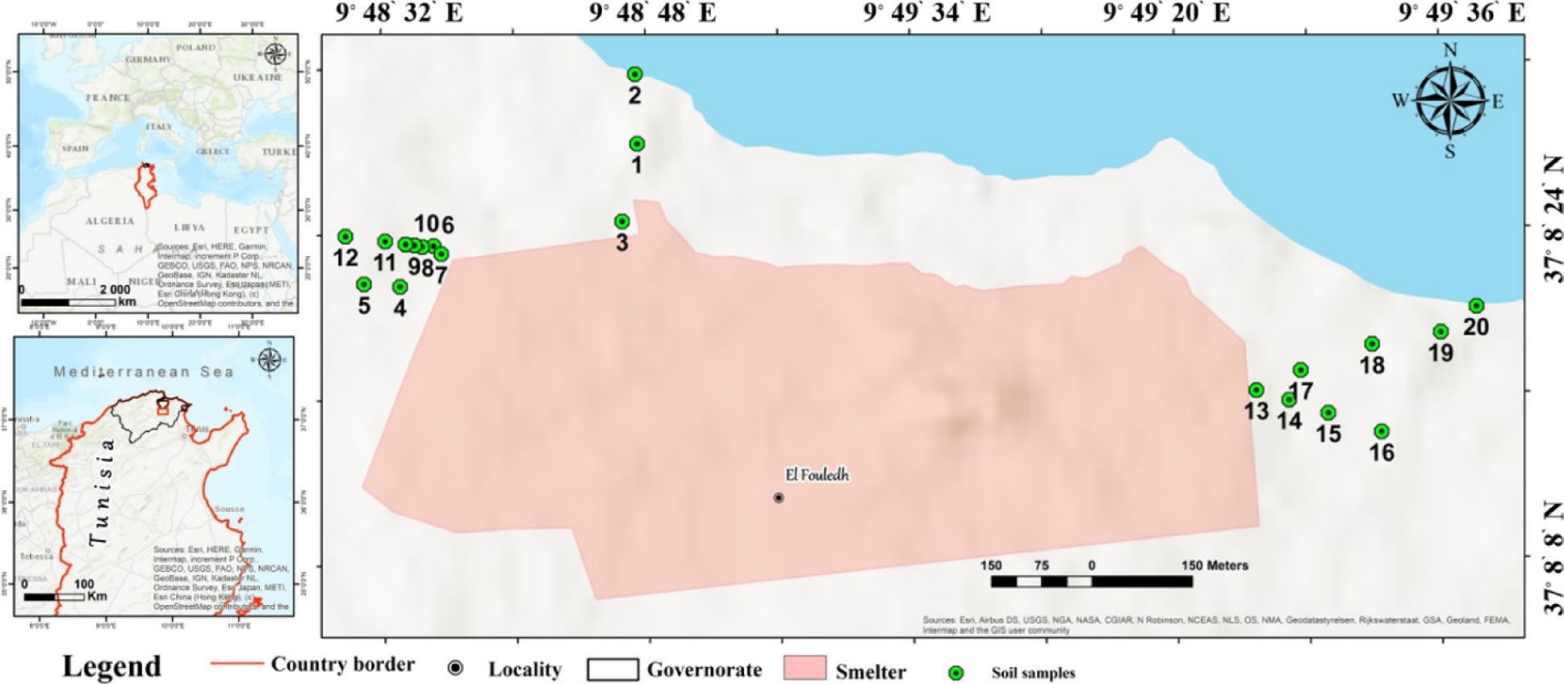
Coordinates and locations of the soil samples in the study area.

The steel smelter of Menzel Bourguiba was founded in 1962, and its production process is mainly hydrometallurgical iron. It is situated in the common region of the urban and the rural of the Menzel Bourguiba city, which has a Mediterranean climate with a mean precipitation of 536 mm/year, annual mean temperature of 17.9 °C and a mean evapotranspiration of about 889 mm/year (Fig. 1). The main soils in the districts are alluvial clay, sandy clay, and black clay^31^. The smelter is located on a hill surrounded by agricultural land. For a long time, pollutants (like exhaust gas, domestic sewage, and wastewater) from smelter factory released disorderly, this has caused significant harm to the natural ecological system of the land, and soil pollution had led to the risk of human health of the neighboring.

HMs in agricultural soil were scrutinized to assess the health risks in the area surrounding the steel smelter of Menzel Bourguiba. In November 2023, 20 soil samples (0–20 cm depth) were taken from agricultural soils by using stainless-steel shovel. In accordance with national regulations, the concerned authority must be notified prior to any soil sampling activity. For this study, the Bizerte Soil Directorate was officially informed by telephone, following the established protocol. The Directorate provided logistical support, including a vehicle and an assigned manager, to facilitate access to private agricultural lands in the vicinity of the Menzel Bourguiba steel smelter. Soil samples were stored in a plastics bag, labeled individually, and transferred to the laboratory to remove stones, and plant residues and then crushed with an agate mortar and sieved through a 2-mm mesh. Sample preparation was performed according to the EPA 3051A method.

About 0.5 g of air-dried soil sample was dissolved using a combination of concentrated acid (3 ml HCl + 4 ml HNO_3_ + 1 ml H_2_O_2_ + 0.5 ml HF) and diluted with 3% HNO_3_ to 10 ml for subsequent analysis. Then, the HMs contents (As, Pb, Zn, Fe, Cd, Cu, Cr, and Ni) were analyzed using Atomic Absorption Spectrometer Perkin-Elmer Analyst 200. The analytical precision was within 10% of the variability. All analyses were conducted in triplicate with the standard deviation of ≤ 5%.

The geographic coordinates for each of the 20 sampling locations are provided in Fig. 1. Sampling was conducted at a depth of 0–20 cm to represent the plow layer, which is the primary zone of root uptake for crops and the most relevant layer for direct human exposure through ingestion and dermal contact. Each of the 20 locations represents a distinct composite sample, and no field replicates were taken.

Quality assurance and quality control (QA/QC) were ensured through the analysis of procedural blanks, duplicate samples, and a certified reference material. The recovery rates for all analyzed metals were within the acceptable range of 90–110%, confirming the accuracy of the analytical results.

### Soil contamination level assessment

To discern the degree of contamination in the soil around the steel smelter of Menzel Bourguiba, the geoaccumulation index (Igeo), the contamination factor (CF), and the enrichment factor (EF) were assessed. For all contamination indices (Igeo, EF, CF, PLI), the background values (Bn) are taken from the Average Shale Value (ASV) of Turekian and Wedepohl, which is appropriate for the fine-grained alluvial soils of the study area^32^. The Upper Continental Crust (UCC) values are presented in Table 1 for comparative context only and are not used in index calculations. This harmonized approach ensures consistency across all pollution indices.

**Table 1:** Descriptive statistics of HMs concentration (mg kg^−1^) in the agricultural soil in the vicinity of Menzel Bourguiba steel smelter.

	As	Pb	Zn	Fe	Cd	Cu	Cr	Ni
Maximum	161.56	364.00	1851.00	63,840	12.00	478.00	538.13	101.00
Minimum	5.10	49.00	159.00	15,750	1.00	8.00	160.70	65.00
Mean	15.97	147.12	480,73	28,136	5.27	55.50	184.85	73.50
SD	34.40	110.76	406.08	11,233.63	2.64	104.16	83.21	7.29
CV (%)	215	75	84	40	50	188	45	10
Kurtosis	19	−1	6	5	1	15	19	11
Skewness	4.4	0.9	2.5	1.9	0.9	3.9	4.5	3.0
UCC^a^	4.8	17	67	39,200	0.09	28	92	47
ASV^b^	13	20	95	47,200	0.3	45	90	68

*SD* standard deviation, *CV* coefficient of variation, *UCC*^a^, upper continental crust values, and *ASV*^b^ average shale volume.

#### Enrichment factor (EF)

The EF evaluates the extent of anthropogenic inputs by comparing analyzed HMs concentrations to natural background levels), and is calculated using Eq. 1^33,34^

1 $${\mathrm{EF}} = \frac{{\left( {\frac{{C_{n} }}{{C_{{{\mathrm{Fe}}}} }}} \right)_{{{\mathrm{sample}}}} }}{{\left( {\frac{{C_{n} }}{{C_{{{\mathrm{Fe}}}} }}} \right)_{{{\mathrm{background}}}} }}$$

where $C_{{\mathrm{n}}}$ sample denotes the concentration of the examined HMs in soil and and $C_{{\mathrm{n}}}$ denotes the concentrations of the references HMs for normalization; Fe sample and Fe background: concentration of Fe in soil and the background environment. According to Saha^35^, EF is classified into five groups (Table S1). Iron (Fe) was selected as the normalizing element for the Enrichment Factor (EF) calculation due to its abundance in the Earth’s crust and its widespread use as a reference element in soil contamination studies to distinguish between natural and anthropogenic sources^35^. Although Fe concentrations can be influenced by smelter activities, the consistent pattern of EF values across the 20 sampling sites demonstrates that Fe remains an appropriate normalizer for this study. Specifically, the clear differentiation between highly enriched elements (Cd and Pb, with mean EF values of 35.48 and 16.04, respectively) and minimally enriched elements (Ni, Cu, and As, with mean EF values < 2.5) indicates that the enrichment factors are effectively capturing the anthropogenic contribution to soil contamination. the measured mean Fe concentration in the study area (28,136 mg kg^−1^) is substantially lower than both the ASV (47,200 mg kg^−1^) and the UCC (39,200 mg kg^−1^) background values. The mean CF for Fe is 0.60 and the mean Igeo for Fe is − 1.42, both indicating that Fe is not enriched relative to background. This is a critical finding: if the smelter were significantly enriching Fe in the surrounding soils, we would expect CF > 1 and Igeo > 0, which is not observed. The absence of Fe enrichment strongly supports the validity of Fe as a normalizing element in this specific context.

#### Geo‑accumulation index (Igeo)

The Igeo was proposed in the 1960s to quantitatively asses HM pollution in soils ^36–38^ and was computed from the following equation (Eq. 2):

2 $$I_{{{\mathrm{geo}}}} = {\mathrm{Log}}_{2} \left( {\frac{{C_{n} }}{{1.5B_{n} }}} \right)$$

where *C*_*n*_* is* the measured concentration of HMs (mg kg^−1^) in the soil samples; and *B*_*n*_ is the background value of the analyzed HMs (mg kg^−1^) in the Earth’s crust. The value of 1.5 is used to account probable variations due to lithogenic influences^39^.

The background values (*B*_n_) of the investigated HMs (Fe: 47,200 mg kg^−1^; Zn: 95 mg kg^−1^; Cr: 90 mg kg^−1^; Ni: 68 mg kg^−1^; Cu: 45 mg kg^−1^; Pb: 20 mg kg^−1^; As: 13 mg kg^−1^; Cd: 0.3 mg kg^−1^)^32^. According to Müller^36^, the ${\mathrm{I}}_{{{\mathrm{geo}}}}$ is classified into seven classes (Table S1).

#### Contamination factor (CF)

The CF was computed to appraise the HMs pollution levels in soil samples (Eq. 3), which involves employing a contamination agent^40^

3 $${\mathrm{CF}} = \frac{{C_{{{\mathrm{metal}}}} }}{{C_{{{\mathrm{background}}}} }}$$

where C_metal_ denotes metal concentration in soil sample; C_background_ denotes background value of that metal. The CF is classified into four groups, itemized Table S1.

#### Pollution load index (PLI)

The PLI gives an evaluation of the overall toxicity status of the soil as a result of the contribution of several metals^13,19,20,41,42^. This parameter is expressed as mentioned in Eq. 4.

4 $${\mathrm{PLI}} = \sqrt[n]{{{\mathrm{(CF}}_{1} \times {\mathrm{CF}}_{2} \times \ldots {\mathrm{CF}}_{3} }})$$

where CF = contamination factor; n = number of metals. According to Tomlinson^38,41,43^, the PLI is classified into four groups (Table S1).

### Health risk assessment

The human health risk assessment model proposed by USEPA^15,44^ was used for the health risk assessment. In view of the physiological differences between risk receptors, the health risks for children and adults were evaluated separately^22,28,29^. In accordance with the established model guidelines, health risks were classified into two categories: non-carcinogenic risk and carcinogenic risk. In order to ascertain the risk assessment process arising from HMs in analyzed soil, the following equations (Eqs. 5–7) were applied^15,44^.

5 $${\mathrm{ADI}}_{{{\mathrm{ing}}}} = \frac{{{\mathrm{C}}_{{{\mathrm{soil}}}} \times {\mathrm{IngR}} \times {\mathrm{EF}} \times {\mathrm{ED}}}}{{{\mathrm{BW}} \times {\mathrm{AT}}}} \times 10^{ - 6}$$

6 $${\mathrm{ADI}}_{{{\mathrm{inh}}}} = \frac{{{\mathrm{C}}_{{{\mathrm{soil}}}} \times {\mathrm{InhR}} \times {\mathrm{EF}} \times {\mathrm{ED}}}}{{{\mathrm{BW}} \times {\mathrm{AT}} \times {\mathrm{PEF}}}}$$

7 $${\mathrm{ADI}}_{{{\mathrm{derm}}}} = \frac{{{\mathrm{C}}_{{{\mathrm{soil}}}} \times {\text{ SA }} \times {\text{ AF }} \times {\text{ ABS }} \times {\text{EF }} \times {\text{ ED}}}}{{{\text{BW }} \times {\text{ AT}}}} \times 10^{ - 6}$$

where ADI_ing_, ADI_inh_ and ADI_derm_ represent the average daily exposures of soil HMs through ingestion, inhalation, and dermal exposure pathways, respectively. C_soil_ is the concentration of HMs in the soil. Table S2 displays the description and values of the parameters used in Eqs. (5), (6) and (7).

The non-carcinogenic hazard quotients (*HQ*) were then computed by dividing the estimated exposure dose to the daily reference dose (*RfD*) (Table S3), and the total hazard index (*HI*) was the sum of the *HQ* from the different pathways. HQ and HI can be expressed mathematically using Eqs. (8–11).

8 $$HQ_{ing} = \frac{{ADI_{ing} }}{{RfD_{ing} }}$$

9 $$HQ_{inh} = \frac{{ADI_{inh} }}{{RfD_{inh} }}$$

10 $$HQ_{derm} = \frac{{ADI_{derm} }}{{RfD_{derm} }}$$

11 $$HI = HQ_{ing} + HQ_{inh + } HQ_{derm}$$

HQ or HI values lower than 1 denotes no significant non-carcinogenic effects, whereas values higher than 1 indicates an adverse non-carcinogenic risk^45^.

To appraisal the values of carcinogenic risk and the total carcinogenic risk (TCR) for the three pathways of exposure, the following equations (Eqs. 12–15) are used.

12 $$CR_{ing} = ADI_{ing} \times CSF_{ing}$$

13 $$CR_{inh} = ADI_{inh} \times CSF_{inh}$$

14 $$CR_{derm} = ADI_{derm} \times CSF_{derm}$$

15 $$TCR = CR_{ing} + CR_{inh} + CR_{derm}$$

where CSF represents the cancer slope factor. CR and TCR values lower than 1 × 10^−6^, between 1 × 10^−6^ and 1 × 10^−4^, and greater than 1 × 10^−4^ implies insignificant risk, tolerable risk, or unacceptable carcinogenic risk, respectively^45^.

### Monte Carlo simulation and sensitivity analysis

Monte Carlo simulation was implemented to characterize uncertainty and variability in risk estimates using Python for each analysis^18,21–23,46^. The Monte Carlo simulation and Sobol sensitivity analysis were performed using Jupyter Lab (Version 3.6.3) with Python and the following libraries: NumPy (Version 1.23.5 or later) for numerical computations, Pandas (Version 1.5.0 or later) for data manipulation, SciPy (Version 1.9.3 or later) for statistical functions, and SALib (Version 1.4.5 or later) for global sensitivity analysis. Specifically, the saltelli sampler from SALib was used to generate the sample matrix for the Sobol analysis, and the sobol analyzer was used to compute first-order (Si), second-order (Sij), and total-order (STi) sensitivity indices. A total of 10,000 iterations were performed for each Monte Carlo simulation to characterize the uncertainty in health risk estimates. The adequacy of this sample size was verified through convergence analysis, in which output distributions (mean, standard deviation, and percentiles) were compared for simulations with 5000, 10,000, and 15,000 iterations. The convergence analysis demonstrated that output statistics stabilized at 10,000 iterations, with differences of less than 2% between the 10,000 and 15,000 iteration scenarios (Table S1). This analysis confirmed that 10,000 iterations were sufficient to achieve convergence in the output distributions without requiring additional computational burden. Probability distributions were assigned to key input parameters based on literature values, and measured data. Parameter distributions included in previous equations. Monte Carlo simulations were conducted separately for children and adults, generating probability distributions for HQ, HI, and TCR values. Output distributions were characterized using percentile values (5th, 50th, 95th percentiles) and exceedance probabilities for relevant risk thresholds.

Global sensitivity analysis was performed using Sobol indices to identify parameters contributing most significantly to uncertainty in risk estimates. First-order Sobol indices (S₁) quantify the direct contribution of individual parameters to output variance, while total-order indices (ST) capture total effects including parameter interactions. Sobol indices were calculated for HI values using variance decomposition methods implemented in Python. Parameters with high Sobol indices (> 0.1) were identified as critical for risk assessment accuracy and prioritized for uncertainty reduction efforts^47^.

It is important to note that Monte Carlo simulation generates probability distributions for risk estimates rather than deterministic outcomes. Exceedance probabilities indicate the likelihood that a risk threshold is exceeded under the assumed parameter distributions, not the certainty that all exposed individuals will experience adverse health effects.

### Multivariate statistical analysis

The research used Hierarchical cluster analysis (HCA) with dendrogram visualization and Principal Component Analysis (PCA) to identify and separate the sources of metal contamination in soil samples. The HCA analysis used Ward’s linkage method with Euclidean distance to create dendrograms which show how different metals relate to each other through their concentration patterns and spatial distribution^48^. The dendrogram structure shows how metals with similar geochemical behaviors and shared emission sources or geochemical processes group into distinct clusters.

The PCA method converted the extensive multi-element dataset into uncorrelated principal components which explained the highest amount of data variance. The z-score normalization method standardized the metal concentration data before PCA to remove scale-related differences between elements. The Kaiser criterion was used to select components with eigenvalues greater than 1 and varimax rotation was applied to improve the interpretability of factor loadings^23,46,49,50^. The PCA loadings showed which metals were most influential in each principal component which helped researchers identify specific pollution origins including industrial activities and traffic emissions and natural weathering processes.

The combination of HCA with PCA established a strong method to identify between natural geogenic origins and human-made pollution sources. The natural elements in the soil tend to group with each other through components that show high loadings for natural variations while anthropogenic metals create distinct clusters with unique principal component patterns^28^. The combined method shows excellent results in environments with various pollution sources because it allows researchers to identify different pollution sources through their unique metal composition.

The statistical analysis for all calculations with *p* < 0.05 as the significance threshold. The dendrogram cut-off distance selection followed the largest fusion coefficient increase while PCA component interpretation used factor loadings above 0.5 for strong associations and between 0.3 and 0.5 for moderate associations^51^.

## Results and discussion

### Heavy metal concentrations in agricultural soils

Table 1 presents comprehensive descriptive statistics for heavy metal(loid) concentrations in soil samples collected from the study area. The mean concentrations of the eight analyzed metals (excluding Fe) ranked from highest to lowest are: Zn (480.73 mg kg^−1^) > Cr (184.85 mg kg^−1^) > Pb (147.12 mg kg^−1^) > Ni (73.5 mg kg^−1^) > Cu (55.50 mg kg^−1^) > As (15.97 mg kg^−1^) > Cd (5.27 mg kg^−1^). Iron (Fe) concentrations averaged 28,136 mg kg^−1^, substantially higher than other metals but below natural crustal abundances (discussed below).

The coefficient of variation (CV) reveals substantial spatial heterogeneity in metal distribution across the study area. With the exception of nickel (CV = 10%), all metals exhibited high variability (CV > 30%), indicating non-uniform contamination patterns. Arsenic showed the highest variability (CV = 215%), followed by copper (CV = 188%), iron (CV = 40%), zinc (CV = 84%), and lead (CV = 75%). This high variability reflects the influence of anthropogenic activities and proximity-dependent contamination, with the most heavily contaminated areas located adjacent to the smelter facility.

The distribution characteristics of the metals reveal important patterns in contamination. Arsenic, zinc, iron, cadmium, copper, and nickel exhibited positive kurtosis values, indicating relatively peaked (leptokurtic) distributions concentrated around the mean. In contrast, lead displayed negative kurtosis (− 1), indicating a relatively flat (platykurtic) distribution. Skewness analysis revealed that most metals displayed positive skewness (rightward tail), with arsenic showing the highest skewness (4.4), followed by chromium (4.5), copper (3.9), nickel (3.0), zinc (2.5), and iron (1.9). Cadmium and lead showed lower skewness values (0.9), indicating more symmetric distributions. These distribution characteristics are consistent with contamination from a point source (the smelter facility), where metals are progressively diluted with distance from the source.

Table 1 presents a comparison of measured heavy metal(loid) concentrations with benchmark values from the upper continental crust (UCC) and average shale value (ASV), which represent natural background levels. Iron concentrations were slightly lower than both UCC (39,200 mg kg^−1^) and ASV (47,200 mg kg^−1^) values, indicating that iron enrichment is not a characteristic of the contamination. In contrast, all other analyzed metals showed substantial enrichment relative to background values.

Cadmium exhibited the most severe enrichment, with mean concentrations approximately 59 times higher than the UCC value and 18 times higher than the ASV value. Lead and zinc also showed substantial enrichment, with mean concentrations 8.7 times and 7.2 times higher than the UCC, respectively, and 7.4 times and 5.1 times higher than the ASV, respectively. Arsenic concentrations were 3.3 times higher than the UCC but only 1.2 times higher than the ASV, indicating moderate enrichment. Chromium and copper showed moderate enrichment, with mean concentrations 2.0 times higher than the UCC and 2.0 times and 1.2 times higher than the ASV, respectively. Nickel displayed minimal enrichment, with mean concentrations 1.6 times higher than the UCC and 1.1 times higher than the ASV.

The consistent enrichment of multiple metals across the study area, particularly the extreme enrichment of cadmium and lead (primary byproducts of steel smelting), combined with the spatial heterogeneity of contamination and the lack of iron enrichment, provides strong evidence that the soil contamination is primarily anthropogenic in origin and directly attributable to historical and ongoing operations at the Menzel Bourguiba steel smelter.

### Soil contamination assessment using multiple pollution indices

**Fig. 2:**
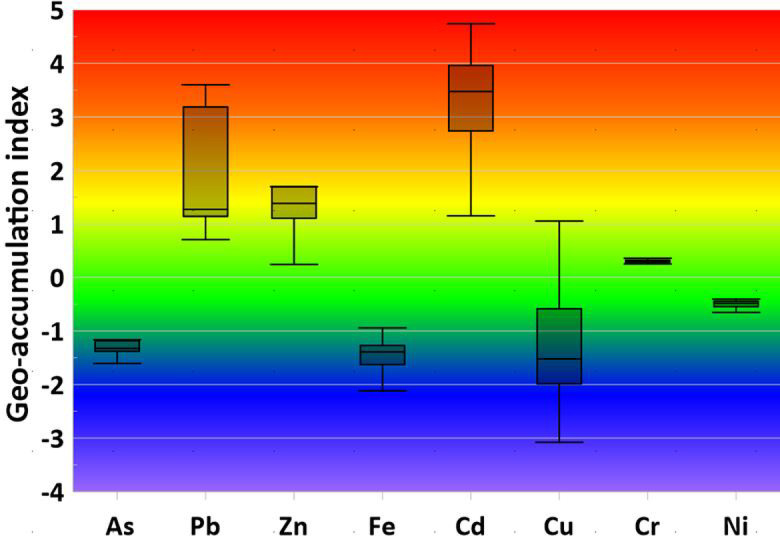
Geoaccumulation index of HMs in the soil near Menzel Bourguiba smelter.

**Fig. 3:**
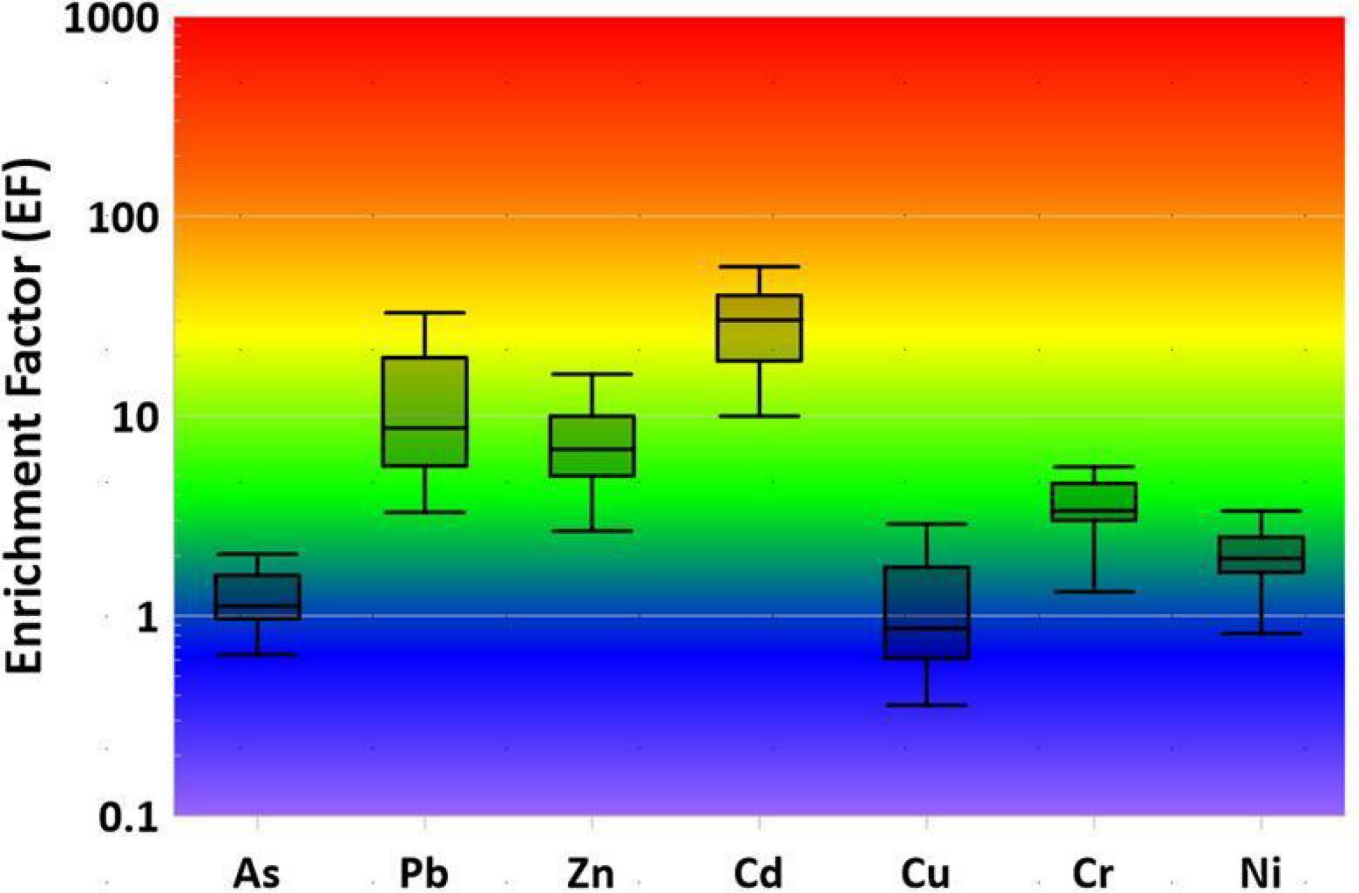
Enrichment factor of HMs in the soil near Menzel Bourguiba smelter.

Multiple geochemical pollution indices were employed to comprehensively assess the degree and spatial distribution of heavy metal(loid) contamination in agricultural soils near the Menzel Bourguiba steel smelter. The results from four complementary indices (geo-accumulation index (Igeo), enrichment factor (EF), contamination factor (CF), and pollution load index (PLI)) are presented in Figs. 2 and 3 and Table 2, providing a multifaceted evaluation of soil contamination.

**Table 2:** Contamination factor (CF) values and Pollution load index (PLI) of HMs in the soil near Menzel Bourguiba smelter.

CF									PLI
	As	Pb	Zn	Fe	Cd	Cu	Cr	Ni	
Min	0.39	2.45	1.67	0.33	3.33	0.18	1.79	0.96	1.08
Max	12.43	18.20	19.48	1.35	40	10.62	5.98	1.49	3.48
Mean	1.23	7.36	5.06	0.60	17.56	1.23	2.05	1.08	2.01

#### Cadmium and lead as primary contaminants

Cadmium (Cd) and lead (Pb) emerged as the primary contaminants across all four pollution indices. Igeo values for cadmium ranged from 1.15 to 4.74 (mean = 3.36), indicating “very high to extreme contamination” (Igeo > 3), with 100% of samples exceeding the Igeo threshold of 1. Lead showed Igeo values ranging from 0.71 to 3.60 (mean = 1.93), with 95% of samples exceeding Igeo = 1, indicating “moderate to very high contamination”. The enrichment factor (EF) results were consistent with the Igeo findings: cadmium displayed extreme enrichment (mean EF = 35.48, with a maximum of 115.40 at sample S3), while lead showed substantial enrichment (mean EF = 16.04). The contamination factor (CF) results further confirmed the severity of Cd and Pb contamination, with both metals showing high contamination levels (mean CF = 17.6 for Cd and 7.4 for Pb). These consistent findings across all three indices (Igeo, EF, CF) indicate that cadmium and lead are the dominant contaminants in the study area and are the primary drivers of overall soil contamination.

#### Zinc as a secondary contaminant

Zinc (Zn) was identified as a secondary contaminant, showing substantial enrichment and contamination across multiple indices. Igeo values for zinc ranged from 0.16 to 3.70 (mean = 1.44), with 75% of samples exceeding Igeo = 1, indicating "moderate to very high contamination." The EF results showed substantial enrichment (mean EF = 9.61), with values primarily falling in the 3rd enrichment class (20 ≤ EF < 40). The CF results indicated moderate contamination (mean CF = 5.1), placing zinc as the third most contaminated metal after cadmium and lead. These findings confirm that zinc is a significant but secondary contaminant in the study area.

#### Arsenic, chromium, and copper as tertiary contaminants

Arsenic (As), chromium (Cr), and copper (Cu) showed moderate contamination levels across the indices. Arsenic Igeo values ranged from − 1.93 to 3.05 (mean = − 1.07), with only 5% of samples exceeding Igeo = 1, indicating predominantly “uncontaminated to moderately contaminated” conditions. However, the EF values for arsenic (mean = 1.32) indicated minor enrichment, and the CF values (mean = 1.2) indicated moderate contamination. Chromium showed Igeo values ranging from 0.25 to 1.99 (mean = 0.38), with only 5% of samples exceeding Igeo = 1, indicating predominantly “uncontaminated to slightly contaminated” conditions. The EF results showed moderate enrichment (mean EF = 3.90), and CF values indicated moderate contamination (mean CF = 2.0). Copper displayed Igeo values ranging from − 3.08 to 2.82 (mean = − 1.26), with only 10% of samples exceeding Igeo = 1, indicating predominantly “uncontaminated” conditions. However, the EF results showed moderate enrichment (mean EF = 1.33), and CF values indicated moderate contamination (mean CF = 1.2). The discrepancy between Igeo and EF/CF results for these elements may reflect differences in the reference values used by each index and the specific characteristics of these metals in the study area.

#### Nickel and Iron

Nickel (Ni) and iron (Fe) showed no significant contamination across all indices. Nickel Igeo values ranged from − 0.65 to − 0.01 (mean = − 0.48), indicating “uncontaminated” conditions with no samples exceeding Igeo = 1. The EF results confirmed minimal enrichment (mean EF = 2.00), and CF values indicated minimal contamination (mean CF = 1.1). Iron Igeo values ranged from − 2.17 to − 0.15 (mean = − 1.42), indicating “uncontaminated” conditions. The EF results showed no enrichment (mean EF < 1), and CF values indicated no contamination (mean CF = 0.7), confirming that iron concentrations in the study area are consistent with natural background levels and are not enriched by smelter activities.

#### Overall contamination assessment

The pollution load index (PLI), which integrates the contamination factors of all analyzed metals, provided an overall measure of soil contamination. The mean PLI value for the study area was 1.95, indicating moderate overall soil contamination. Since the PLI > 1, the results confirm that the study area is contaminated by heavy metals. The spatial distribution of PLI values (Fig. 3) reveals a clear pattern of decreasing contamination with distance from the smelter facility, consistent with the expected behavior of a point source of contamination. The highest PLI values were observed in samples collected immediately adjacent to the smelter (samples S1–S5), while lower values were observed in samples collected at greater distances (samples S15–S20).

The consistent findings across all four pollution indices provide strong evidence that the soil contamination in the study area is primarily anthropogenic in origin and directly attributable to historical and ongoing operations at the Menzel Bourguiba steel smelter. The dominance of cadmium and lead as primary contaminants is consistent with the known emission profiles of steel smelting operations, where these metals are major byproducts of the hydrometallurgical iron processing technology employed at the facility. The secondary and tertiary contaminants (Zn, As, Cr, Cu) are also consistent with typical smelter emissions. The absence of enrichment for iron and nickel further supports the conclusion that the contamination is smelter-derived, as these metals are not preferentially enriched by smelting operations. The spatial pattern of contamination (highest near the smelter, decreasing with distance) is consistent with atmospheric deposition and soil transport processes, confirming that the Menzel Bourguiba smelter is the primary source of heavy metal contamination in the study area.

### Non-carcinogenic health risk assessment

#### Hazard quotient (HQ) analysis

**Fig. 4:**
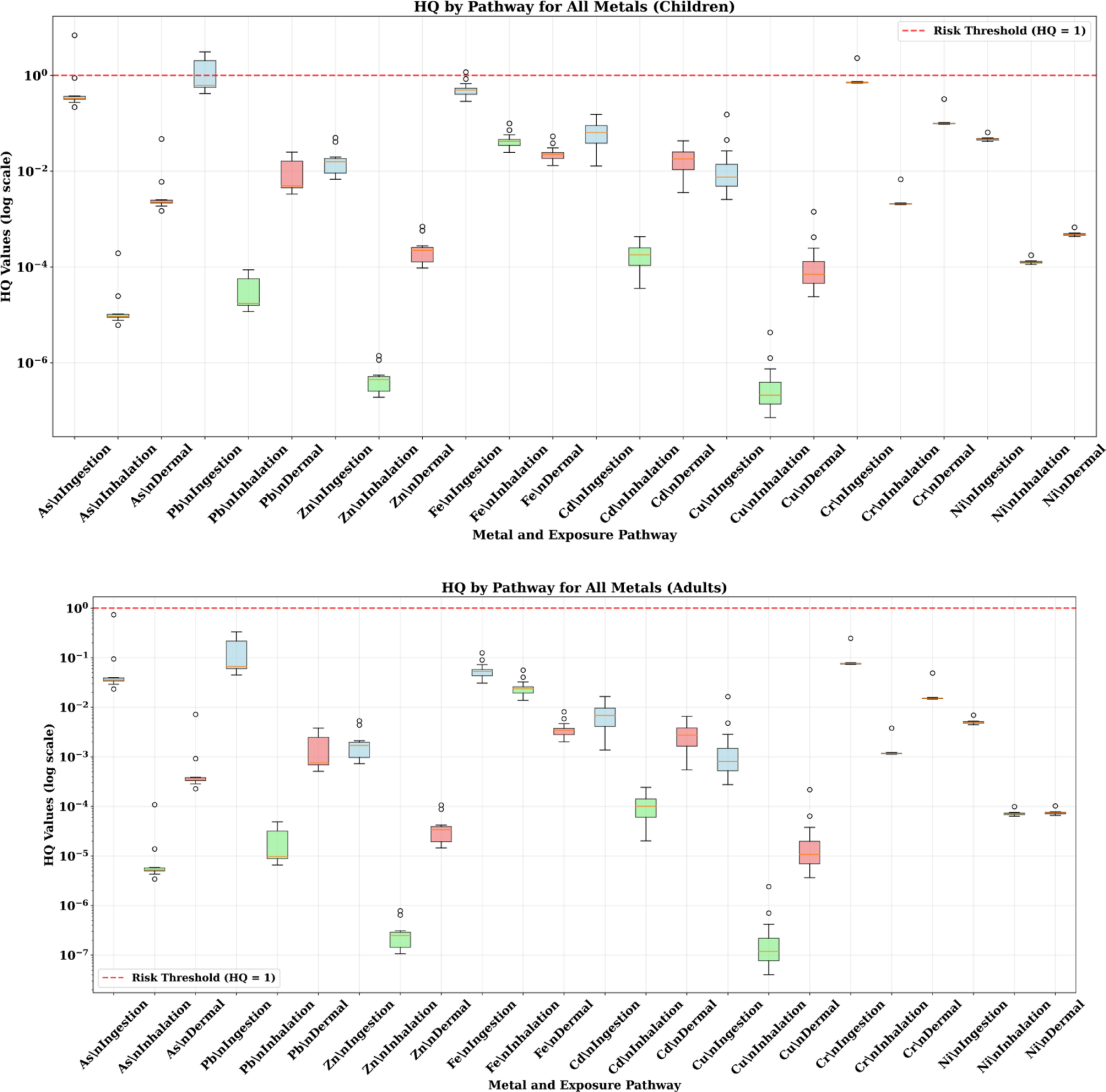
The results of HQ value in adult and children through different pathway exposure associated with heavy metals in the soil samples.

The deterministic HQ analysis revealed significant differences between children and adults, with children showing consistently higher risk levels across all metals and exposure pathways (Fig. 4). For children, the ingestion pathway dominated non-carcinogenic risks, with arsenic showing the highest individual HQ values (median: 0.08, range: 0.03–0.15), followed by iron (median: 0.04, range: 0.02–0.08). While individual HQ values remained below the safety threshold of 1.0, the cumulative effect across multiple metals raised significant concerns.

Adults demonstrated substantially lower HQ values, typically 50–100 times lower than children for equivalent exposure scenarios. This difference primarily reflects physiological factors including higher body weights and lower soil ingestion rates in adults. The inhalation pathway contributed minimally to non-carcinogenic risks for both age groups, while dermal contact showed intermediate contribution levels.

The spatial variability in HQ values reflected the heterogeneous contamination patterns, with some sampling locations showing HQ values approaching the safety threshold. This variability underscores the importance of site-specific risk assessment and the potential for localized areas of elevated risk within the broader study area.

#### Hazard index (HI) analysis—critical findings

**Fig. 5:**
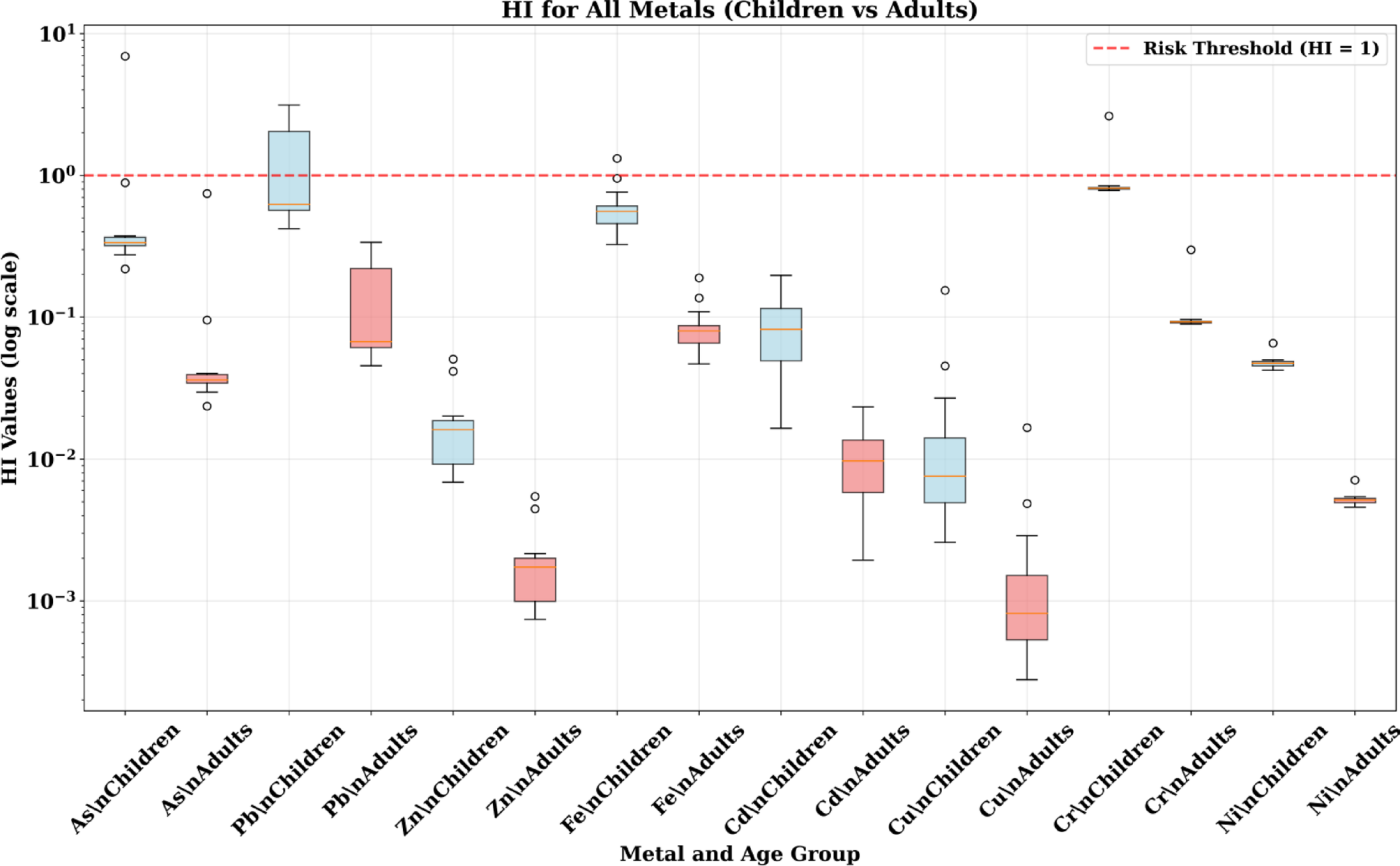
The results of HI value in adult and children through different pathway exposure associated with heavy metals in the soil samples.

The Hazard Index analysis revealed the most concerning findings for non-carcinogenic health risks, particularly for children (Fig. 5). Lead exposure in children showed HI values ranging from 0.5 to 3.0, with median values of approximately 1.2–1.5, clearly exceeding the safety threshold of HI = 1. This finding indicates that the majority of children in the study area face unacceptable non-carcinogenic health risks from lead exposure alone.

Iron also contributed significantly to non-carcinogenic risks in children, with HI values ranging from 0.3 to 1.5 (median: ~ 0.6), with some samples approaching or exceeding the safety threshold. Arsenic showed moderate risk levels with HI values of 0.2–0.4 (median: ~ 0.3), remaining below but approaching concerning levels.

For adults, all HI values remained well below the safety threshold, with lead showing the highest values (HI: 0.05–0.25) but still within acceptable ranges. This dramatic difference between age groups emphasizes the particular vulnerability of children to heavy metal exposure and the critical need for age-specific protection measures.

The exceedance of HI = 1 for lead in children represents a clear public health concern requiring immediate attention. Lead exposure in children is associated with neurodevelopmental effects, reduced IQ, behavioral problems, and impaired cognitive development, even at relatively low exposure levels (Lanphear et al., 2018). The finding that median HI values exceed safety thresholds indicates that the majority of children in the area likely face unacceptable health risks.

### Carcinogenic health risk assessment

#### Cancer risk (CR) by pathway

**Fig. 6:**
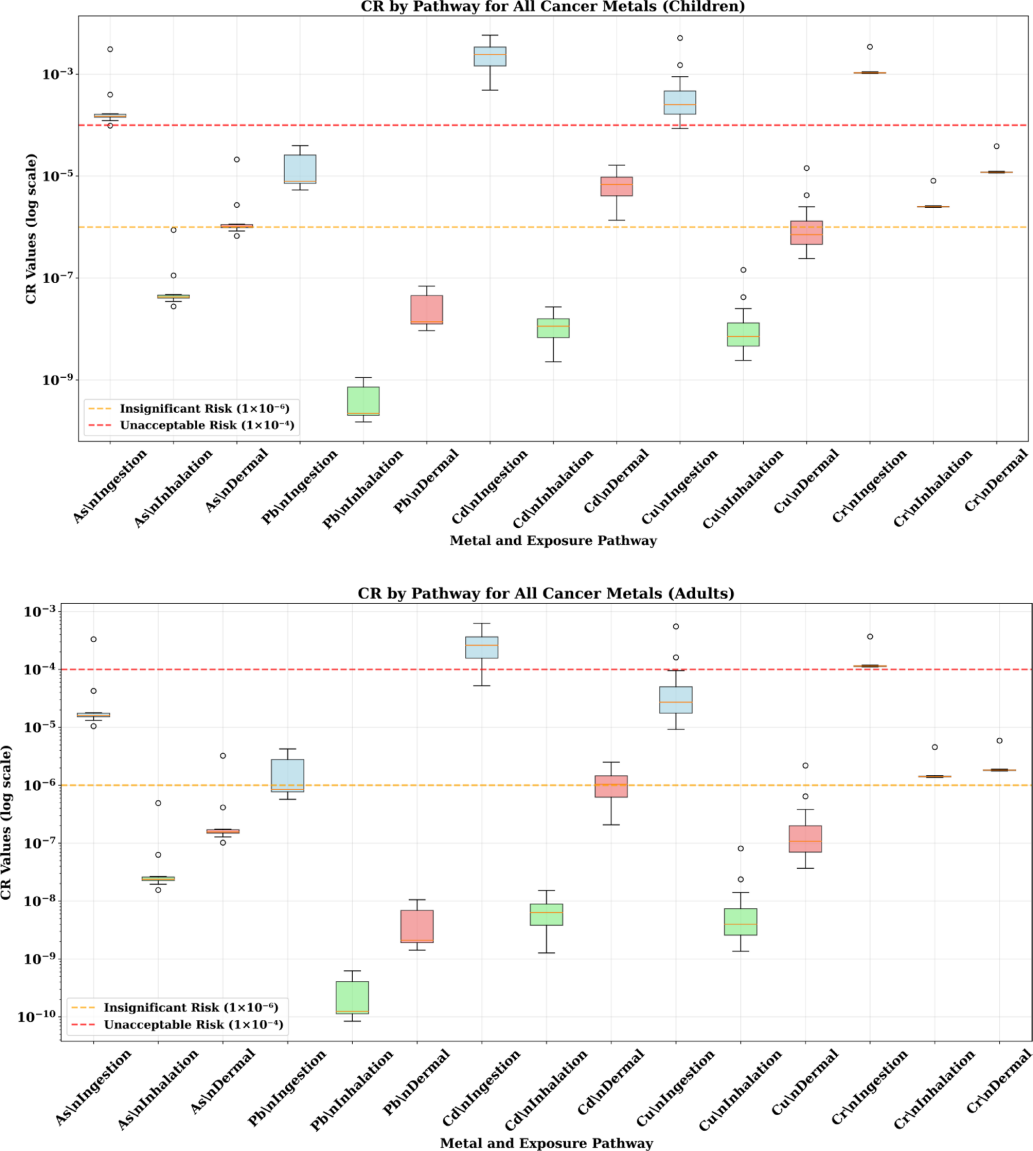
The results of CR value in adult and children through different pathway exposure associated with heavy metals in the soil samples.

The carcinogenic risk assessment revealed elevated cancer risks, particularly for children exposed through the soil ingestion pathway (Fig. 6). Cadmium ingestion showed the highest cancer risk values, with CR values ranging from 1 × 10^−3^ to 3 × 10^−3^ for children, representing 10–30 times the acceptable risk threshold of 1 × 10^−4^. These levels indicate cancer risk values substantially exceeding regulatory thresholds and warrant targeted intervention strategies. Chromium ingestion also posed elevated cancer risks for children, with CR values of 3 × 10^−4^ to 1 × 10^−3^, representing 3–10 times the acceptable threshold. Arsenic ingestion showed CR values of 2 × 10^−4^ to 5 × 10^−4^, representing 2–5 times the acceptable level. The simultaneous exceedance of cancer risk thresholds for multiple carcinogenic metals creates a cumulative cancer risk from multiple exposure sources for the pediatric population.

Adults also faced significant cancer risks, though generally lower than children. Cadmium ingestion in adults showed CR values of 1 × 10^−4^ to 5 × 10^−4^, still exceeding acceptable levels by factors of 1–5. Chromium and arsenic showed lower but still concerning cancer risk levels for adults.

The dominance of the ingestion pathway for cancer risk reflects the high bioavailability of metals through gastrointestinal absorption and the relatively high soil ingestion rates, particularly in children. Inhalation and dermal pathways contributed less significantly to cancer risks but still represented meaningful exposure routes requiring consideration in comprehensive risk management strategies.

#### Total cancer risk (TCR)—cumulative cancer burden

**Fig. 7:**
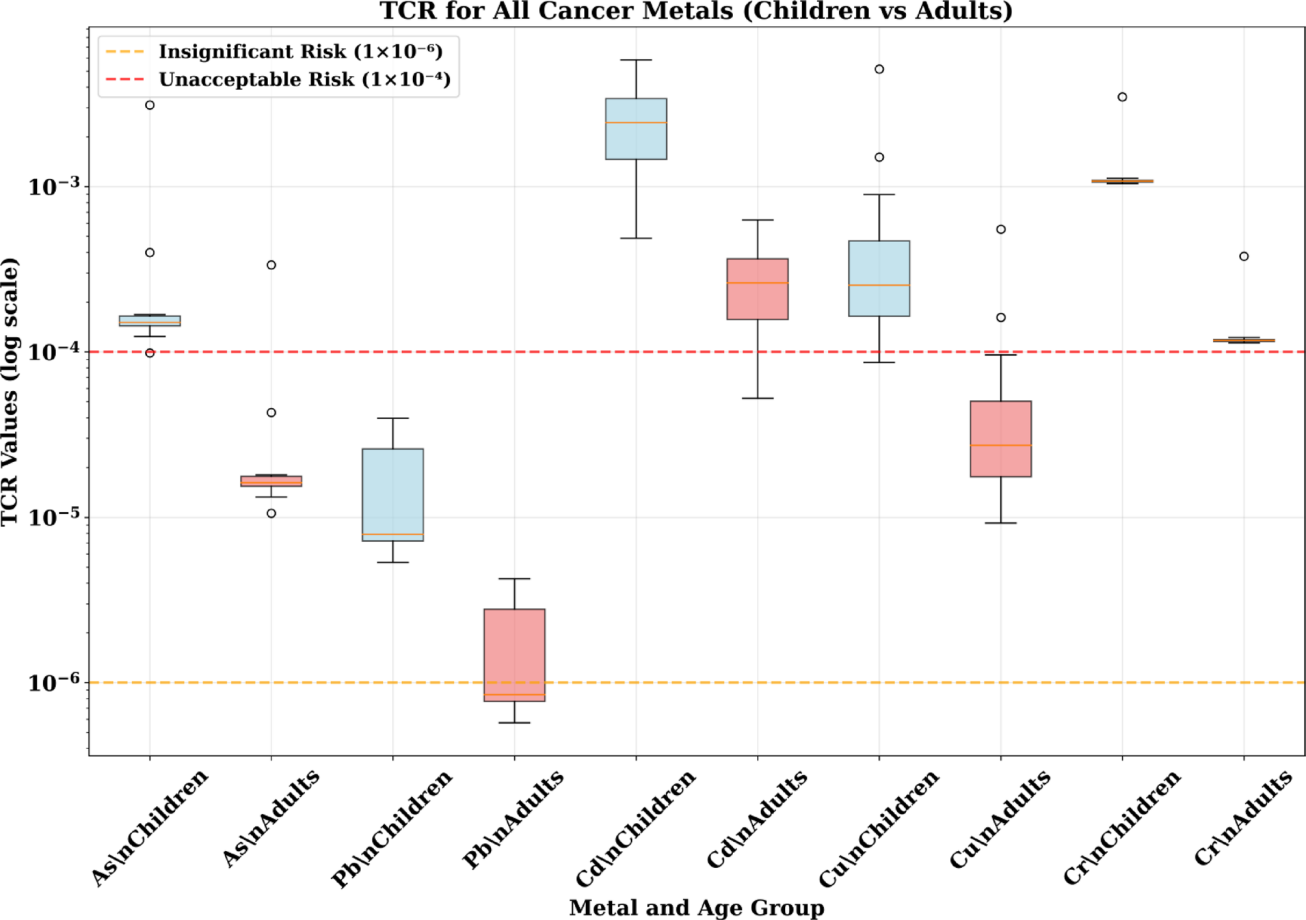
The results of TCR value in adult and children through different pathway exposure associated with heavy metals in the soil samples.

The Total Cancer Risk analysis, representing the cumulative cancer burden from all exposure pathways, revealed the most notable findings of the entire assessment (Fig. 7). For children, cadmium showed TCR values ranging from 1 × 10^−3^ to 4 × 10^−3^, representing 10–40 times the acceptable cancer risk level. These TCR values substantially exceed regulatory thresholds and indicate a significant public health concern requiring targeted intervention. Chromium contributed additional cancer risk with TCR values of 2 × 10 ^−4^ to 8 × 10^−4^ (2–8 times acceptable levels), while arsenic showed TCR values approaching or exceeding the acceptable threshold. The cumulative effect of multiple carcinogenic metals creates an elevated cumulative cancer risk that exceeds acceptable regulatory standards.

For adults, cadmium remained the primary cancer concern with TCR values of 1 × 10^−4^ to 6 × 10^−4^, still exceeding acceptable levels by factors of 1–6. While lower than children’s risks, these levels still represent significant public health concerns requiring intervention.

The implications of these cancer risk levels are profound. A TCR of 1 × 10^−3^ suggests that approximately 1 in 1000 exposed individuals may develop cancer attributable to the exposure, while a TCR of 4 × 10^−3^ suggests 4 in 1000 individuals may be affected. For a community of several thousand residents, these risk levels translate to dozens of potential excess cancer cases directly attributable to environmental contamination.

To contextualize the health risks identified in this study, the results were compared with published data from other smelter-affected and industrially contaminated areas. The mean Total Carcinogenic Risk (TCR) for cadmium in children from this study (approximately 2.5 × 10^−3^) substantially exceeds the USEPA’s acceptable cancer risk threshold of 1 × 10^−4^. Comparative analysis with other industrial sites reveals that the cadmium TCR values at Menzel Bourguiba are among the highest reported in the literature. For example, Zheng^16^ reported a mean TCR of 1.2 × 10^−3^ for children near a copper smelter in Yunnan Province, China, while Zhang^52^ documented a mean TCR of 1.8 × 10^−3^ for children in a lead–zinc mining area in Shaanxi Province, China. Similarly, the non-carcinogenic health risk index (HI) for lead in children from this study (median HI = 1.2–1.5) exceeds published values from other contaminated sites, including a median HI of 1.1 documented by Luo^53^ in a lead-contaminated urban area in Guangdong Province, China. These comparisons indicate that the soil contamination at the Menzel Bourguiba site poses health risks that are comparable to or exceed those documented at other heavily contaminated industrial sites globally, warranting urgent intervention strategies.

### Monte Carlo simulation results

The Monte Carlo simulation results for Hazard Quotient (HQ) values in children reveal critical insights into non-carcinogenic health risks across three exposure pathways. The cumulative probability distributions (P5, P50, P95) demonstrate significant pathway-specific variations and concerning risk levels for multiple heavy metals.

#### Non-carcinogenic risk of metals for children

**Fig. 8:**
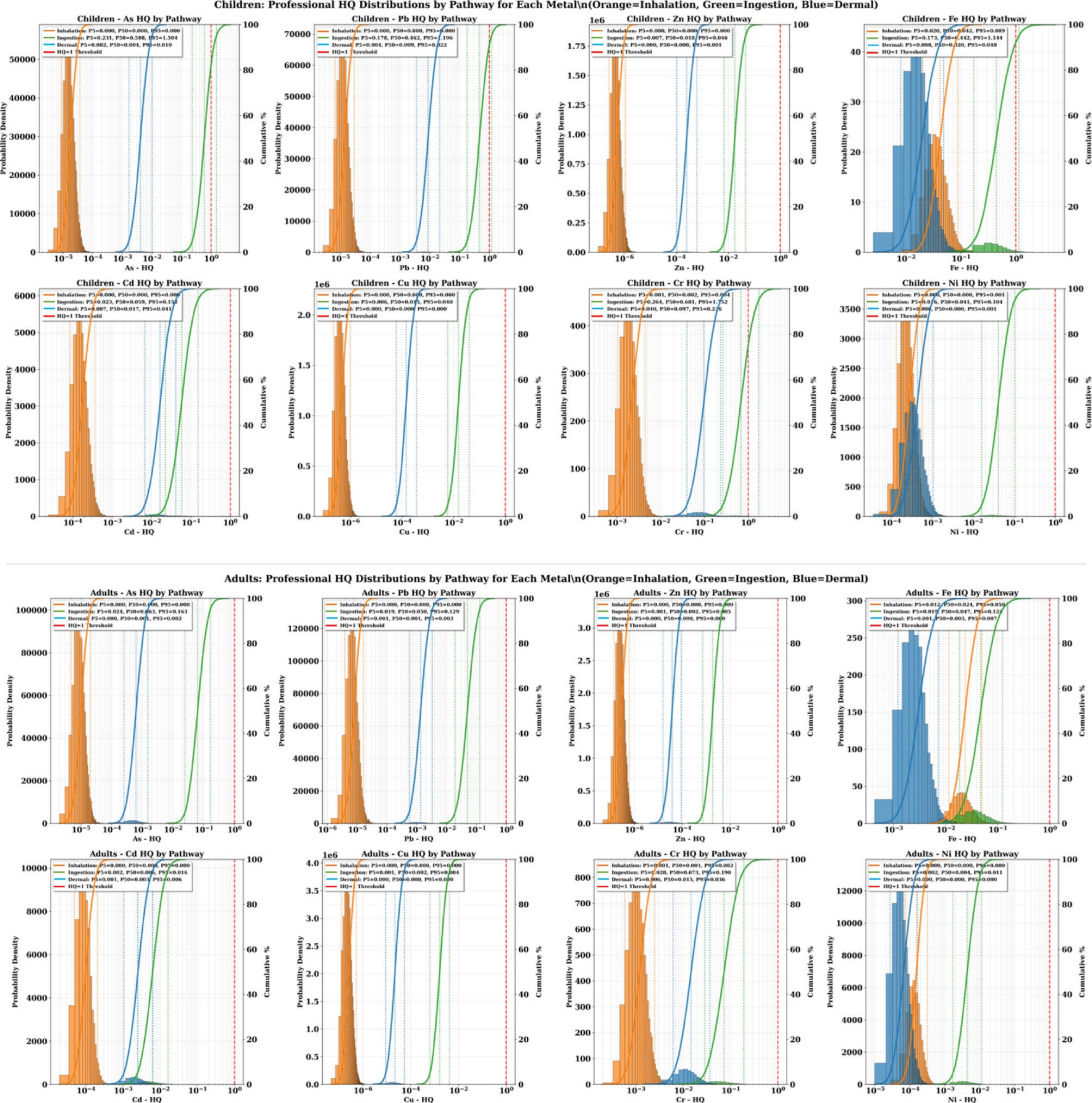
The results of cumulative probability % of HQ value in adult and children through different pathway exposure associated with heavy metals in the soil samples.

The ingestion pathway emerged as the predominant route of exposure, showing the highest HQ values across all metals analyzed. Most critically, several metals demonstrated HQ values approaching or exceeding the safety threshold of 1 at higher percentiles. Chromium exhibited the most concerning ingestion profile, with P95 values reaching 1.752, indicating that 5% of children face HQ levels 75% above the safety threshold. The median (P50) chromium ingestion HQ of 0.681 suggests that half of the exposed children experience HQ levels approaching concerning levels, while even the conservative P5 estimate of 0.264 indicates substantial exposure across the population (Fig. 8).

Arsenic ingestion showed similarly alarming patterns, with P95 values of 1.504 (50% above safety threshold) and median values of 0.588. Lead ingestion demonstrated P95 values of 1.196, approaching the safety threshold, with median values of 0.462. Iron ingestion, while showing lower peak values (P95: 1.144), still approached concerning levels with median values of 0.442. These findings indicate that multiple metals simultaneously contribute to non-carcinogenic health risks through soil ingestion, creating a cumulative exposure burden that significantly exceeds individual metal assessments.

The wide probability distributions for ingestion exposure (large differences between P5 and P95 values) reflect substantial spatial variability in contamination levels, suggesting the presence of contamination hotspots where children face dramatically elevated risks. For chromium, the 6.6-fold difference between P5 and P95 values (0.264 to 1.752) indicates that some children face risks nearly seven times higher than others, emphasizing the critical importance of site-specific risk characterization.

The inhalation pathway consistently showed negligible contributions to non-carcinogenic health risks, with most metals exhibiting HQ values below 10^−5^ across all percentiles. Iron represented the only exception, showing measurable inhalation HQ values (P5: 0.02, P50: 0.042, P95: 0.089), though these remained well below safety thresholds. Chromium showed minimal inhalation risk (P95: 0.004), while all other metals demonstrated essentially negligible inhalation exposure potential.

These findings confirm that airborne exposure to heavy metals, while a theoretical concern near industrial facilities, contributes minimally to actual health risks compared to direct soil contact pathways. The low inhalation HQ values validate the focus on soil ingestion and dermal contact as primary intervention targets for risk reduction strategies.

Dermal contact showed intermediate HQ values between ingestion and inhalation pathways, with chromium again demonstrating the highest risk levels (P95: 0.236, P50: 0.097). Lead and iron showed moderate dermal HQ values (P95: 0.022 and 0.048, respectively), while cadmium exhibited notable dermal exposure potential (P95: 0.041). Other metals showed minimal dermal risk contributions.

The dermal pathway results indicate that skin contact with contaminated soil represents a meaningful but secondary exposure route requiring consideration in comprehensive risk management strategies. The consistent pattern of chromium showing elevated risks across multiple pathways suggests this metal poses particular concern for the pediatric population.

#### Non-carcinogenic risk of metals for children

The Monte Carlo simulation results for adult Hazard Quotient (HQ) values demonstrate a markedly different risk profile compared to children. All cumulative probability distributions (P5, P50, P95) remaining well below the safety threshold of HQ = 1 across all metals and exposure pathways. This finding provides statistical confirmation that adults are effectively protected from non-carcinogenic health effects under current contamination conditions while revealing important insights into pathway-specific exposure patterns and the physiological basis for age-related vulnerability differences.

The ingestion pathway consistently showed the highest HQ values for adults across all metals, maintaining the same pathway hierarchy observed in children but at substantially reduced magnitudes. Chromium exhibited the highest adult ingestion HQ values (P95: 0.190, P50: 0.073), representing approximately 8–10 times lower risk levels than corresponding children’s values. Arsenic ingestion showed the second highest adult HQ levels (P95: 0.163, P50: 0.063), followed by cadmium (P95: 0.152, P50: 0.059) and lead (P95: 0.129, P50: 0.050) (Fig. 8).

The consistent ranking of metals by ingestion risk (Cr > As > Cd > Pb > Fe) mirrors the children’s exposure pattern, confirming that contamination levels and metal-specific toxicity properties drive risk hierarchies independent of age-related physiological factors. However, the dramatic reduction in absolute HQ values (typically 5–tenfold lower than children) demonstrates the critical importance of body weight and ingestion rate differences in determining exposure outcomes.

Iron ingestion showed moderate HQ values (P95: 0.124, P50: 0.047), while other metals including zinc, copper, and nickel demonstrated minimal ingestion risks with P95 values below 0.011. The narrow probability distributions for most metals (small P5–P95 ranges) indicate that physiological parameter variability in adults has less influence on risk estimates compared to children, reflecting the more stable and predictable exposure characteristics of the adult population.

Adult inhalation HQ values remained consistently negligible across all metals, with most showing values below 10^−5^ throughout the probability distribution. Iron represented the only metal with measurable inhalation exposure (P95: 0.050, P50: 0.024), though these values remained far below safety thresholds. Chromium showed minimal inhalation risk (P95: 0.002), while all other metals demonstrated essentially undetectable inhalation exposure potential.

These findings confirm that airborne heavy metal exposure poses minimal non-carcinogenic health risks for adults in the study area, consistent with the children’s inhalation results but at even lower absolute levels. The negligible inhalation contribution validates risk management strategies focused on soil contact pathways rather than air quality interventions for non-carcinogenic health protection.

Dermal contact showed intermediate HQ values between ingestion and inhalation pathways, with cadmium demonstrating the highest dermal risk levels (P95: 0.041, P50: 0.017). Chromium showed moderate dermal HQ values (P95: 0.036, P50: 0.015), while iron and lead exhibited lower dermal exposure potential (P95: 0.007 and 0.003, respectively). Other metals showed minimal or undetectable dermal risk contributions.

The dermal pathway results indicate that skin contact represents a secondary but quantifiable exposure route for adults, contributing approximately 10–20% of total non-carcinogenic risk burden. The consistent pattern of cadmium and chromium showing elevated dermal risks suggests these metals may have enhanced skin penetration characteristics or higher dermal absorption factors.

#### Probability risk of the total hazard index (HI)

**Fig. 9:**
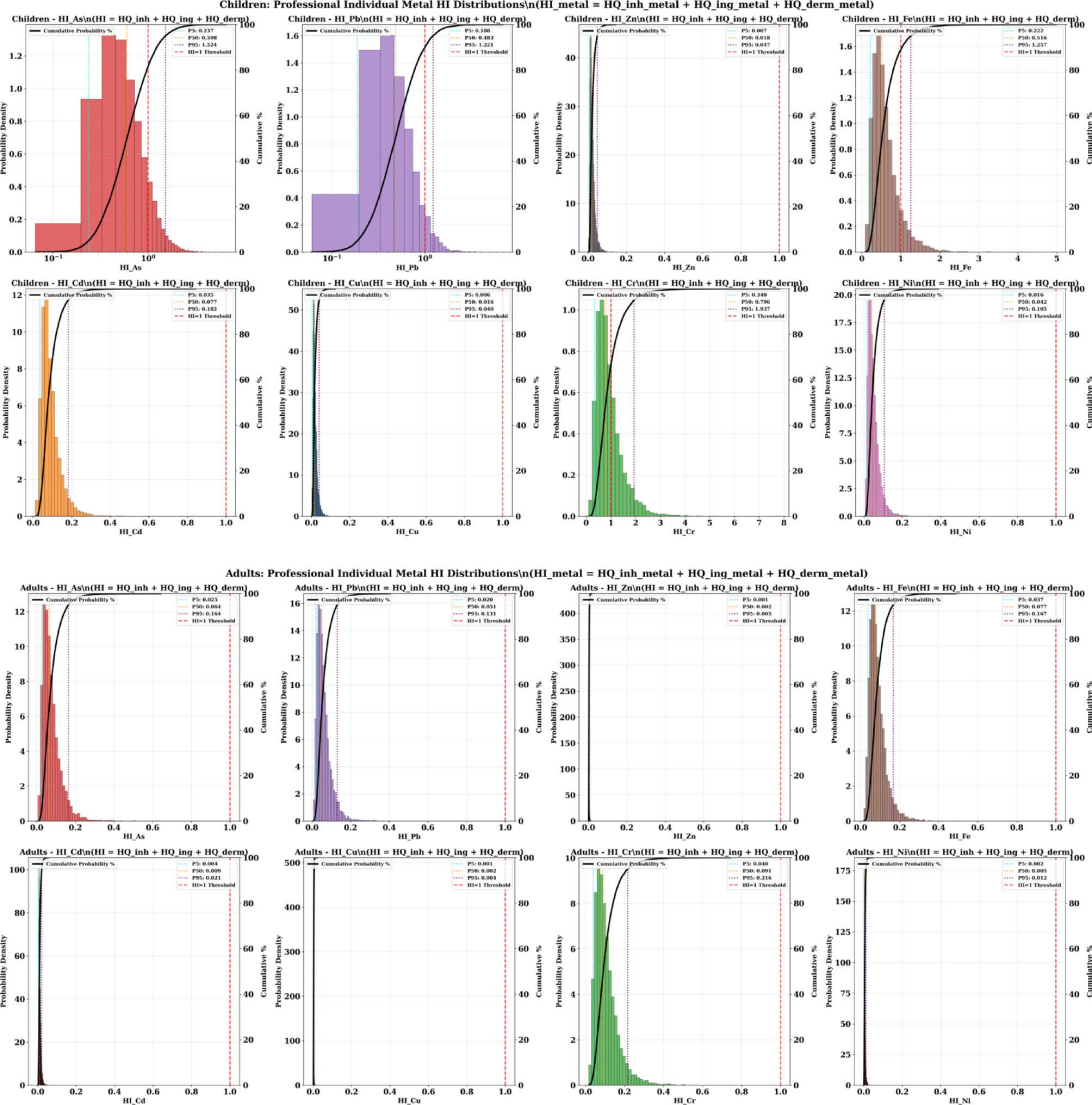
The results of cumulative probability % of HI value in adult and children through different pathway exposure associated with heavy metals in the soil samples.

The Monte Carlo simulation results for Hazard Index (HI) values reveal dramatic age-specific differences in non-carcinogenic health risks, with children facing significant health threats while adults remain safer. The cumulative probability analysis demonstrates that multiple metals pose unacceptable risks to children. Several exceeding the critical safety threshold of HI = 1, while all adult HI values remain well below concerning levels, highlighting the critical importance of age-specific vulnerability assessment (Fig. 9).

The pediatric population faces significant non-carcinogenic health risk, with three metals showing median (P50) HI values exceeding or approaching the safety threshold. Chromium presents the most severe risk profile, with P50 = 0.796 and P95 = 1.937, indicating that 5% of children face chromium exposure risks nearly twice the safety threshold. The probability distribution suggests that approximately 25–30% of children exceed HI = 1 for chromium alone.

Arsenic demonstrates similarly alarming patterns, with P50 = 0.598 and P95 = 1.524, indicating that while median exposure approaches concerning levels, 5% of children face arsenic risks 52% above safety thresholds. Iron shows comparable risk levels (P50 = 0.516, P95 = 1.257), with approximately 15–20% of children likely exceeding the safety threshold.

Lead exposure, while showing a median below the threshold (P50 = 0.483), demonstrates concerning variability with P95 = 1.221, indicating that 5% of children face lead risks 22% above acceptable levels. The cumulative effect of multiple metals simultaneously approaching or exceeding safety thresholds creates an unprecedented cumulative health burden for the pediatric population.

Other metals including cadmium (P95 = 0.182), nickel (P95 = 0.105), zinc (P95 = 0.047), and copper (P95 = 0.040) contribute additional risk burden, though individually remaining below safety thresholds. The simultaneous exposure to multiple metals creates additive health risks that compound the individual metal concerns.

Adult HI values remained below the safety threshold of 1 for all metals and exposure pathways, indicating no significant non-carcinogenic health risk under current exposure conditions. However, this finding should not be interpreted as indicating overall health safety for adults, as the carcinogenic risk assessment demonstrates that adults face significant cancer risks, particularly from cadmium exposure (> 70% probability of TCR exceedance above 1 × 10^−4^). Chromium shows the highest adult risk levels (P50 = 0.091, P95 = 0.216), representing approximately 4–9 times lower values than corresponding children’s exposures. Iron (P95 = 0.167), arsenic (P95 = 0.164), and lead (P95 = 0.131) show moderate but safe exposure levels (Fig. 9).

The dramatic risk reduction in adults reflects the protective effects of higher body weights and lower soil ingestion rates, creating natural physiological barriers to heavy metal exposure. Even under worst-case exposure scenarios (P95 values), adults maintain substantial safety margins, with the highest risk (chromium P95 = 0.216) remaining below the safety threshold.

The comparative analysis reveals striking age-related vulnerability differences, with children showing 4–10 times higher HI values than adults for equivalent environmental exposures. Chromium demonstrates the most dramatic age difference, with children’s P95 values (1.937) being approximately 9 times higher than adults’ P95 values (0.216). Similar patterns emerge for arsenic (9.3 × difference), iron (7.5 × difference), and lead (9.3 × difference). These quantitative differences confirm that body weight and ingestion rate variations create profound age-specific vulnerability patterns that fundamentally alter risk assessment conclusions. The consistent magnitude of protection (5–tenfold) across all metals indicates that age-related physiological differences, rather than metal-specific factors, drive the vulnerability patterns.

The Monte Carlo analysis provides robust statistical evidence supporting immediate pediatric-focused intervention strategies. The multiple metals exceeding safety thresholds in children (chromium, arsenic, iron, lead) create high probability that the majority of children face unacceptable non-carcinogenic health risks from at least one heavy metal exposure.

The comprehensive adult protection demonstrated across all metals indicates that non-carcinogenic risk management strategies can focus resources on pediatric populations without compromising adult health protection. However, the measurable adult exposure levels suggest continued monitoring for potential carcinogenic effects and long-term health impacts not captured in the HI assessment framework.

The wide probability distributions for children (large P5–P95 ranges) indicate substantial spatial variability in pediatric risks. It emphasizes the need for site-specific intervention strategies targeting contamination hotspots while implementing community-wide protection measures. The narrow adult probability distributions suggest more predictable and manageable adult exposure patterns requiring less intensive monitoring and intervention efforts.

### Sobol sensitivity analysis

#### Parameter importance identification

**Fig. 10:**
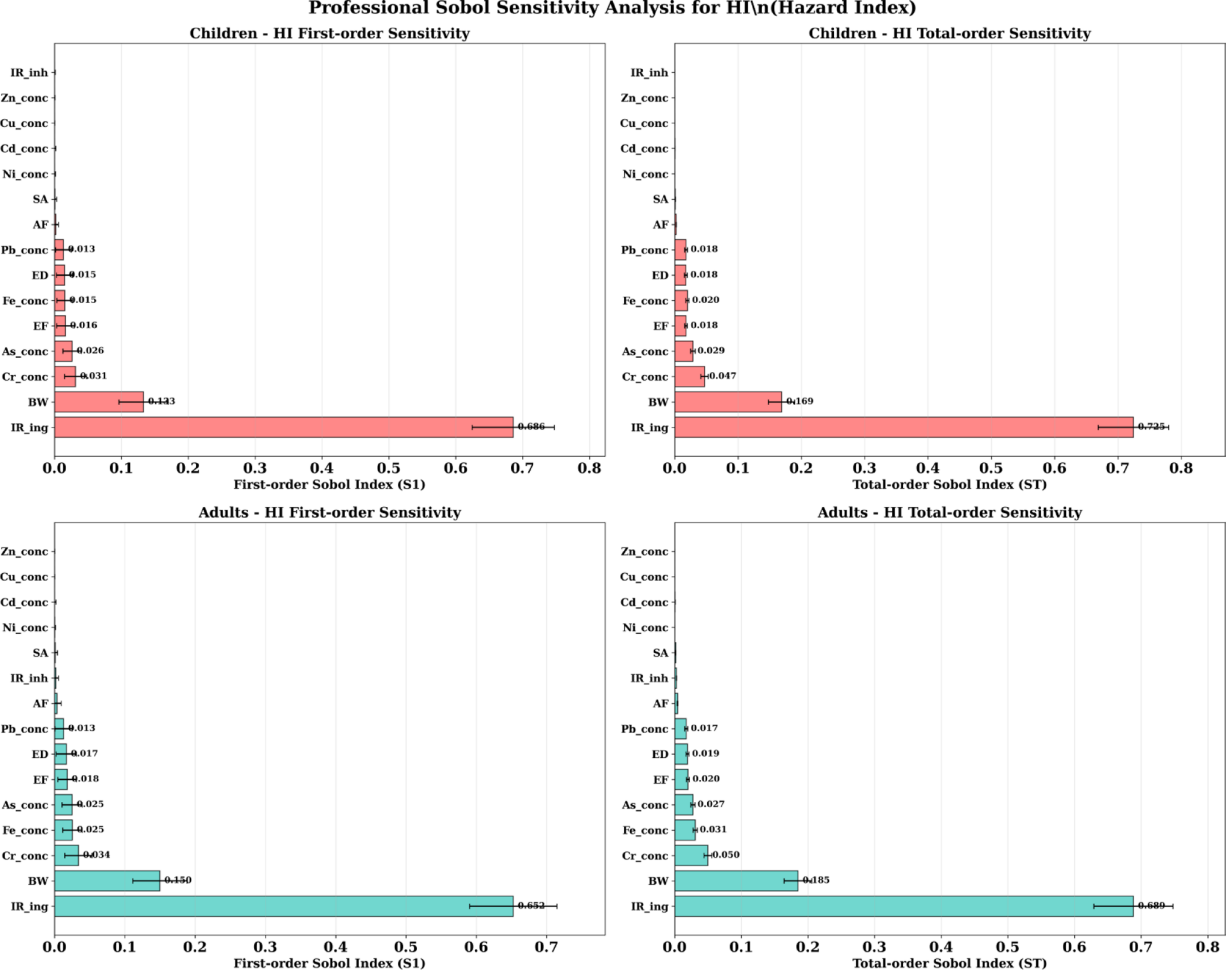
Sobol sensitivity analysis for the parameter importance identification.

The Sobol sensitivity analysis provided crucial insights into the parameters driving uncertainty in the health risk estimates, enabling strategic prioritization of intervention efforts (Fig. 10). For both children and adults, ingestion rate (IR_ing) emerged as the dominant parameter, contributing 68–74% of the total variance in HI calculations. This finding confirms the critical importance of the soil ingestion pathway and highlights behavioral interventions as potentially highly effective risk reduction strategies.

Body weight (BW) represented the second most important parameter, contributing 15–18% of total variance in HI estimates. This finding explains the dramatic differences in risk levels between children and adults and emphasizes the physiological basis for age-specific vulnerability. The high sensitivity to body weight validates the focus on pediatric populations in environmental health risk assessment and intervention planning.

Metal concentrations showed moderate contributions to uncertainty, with chromium (3–5%), iron (2–3%), and arsenic (2–3%) representing the most influential contaminants. This finding indicates that while contamination levels are important, exposure parameters have greater influence on risk estimates than environmental concentrations within the observed range.

### Probabilistic validation of the total cancer risks

The Monte Carlo simulation results for Total Cancer Risk (TCR) values reveal an unprecedented carcinogenic health risk affecting both children and adults. Multiple metals showed probability distributions that dramatically exceed acceptable cancer risk thresholds of 1 × 10^−4^. The analysis demonstrates that children face elevated health cancer risk requiring urgent attention with high probability, while adults also experience significant cancer threats, creating a community-wide carcinogenic risk requiring intervention.

#### Children

**Fig. 11:**
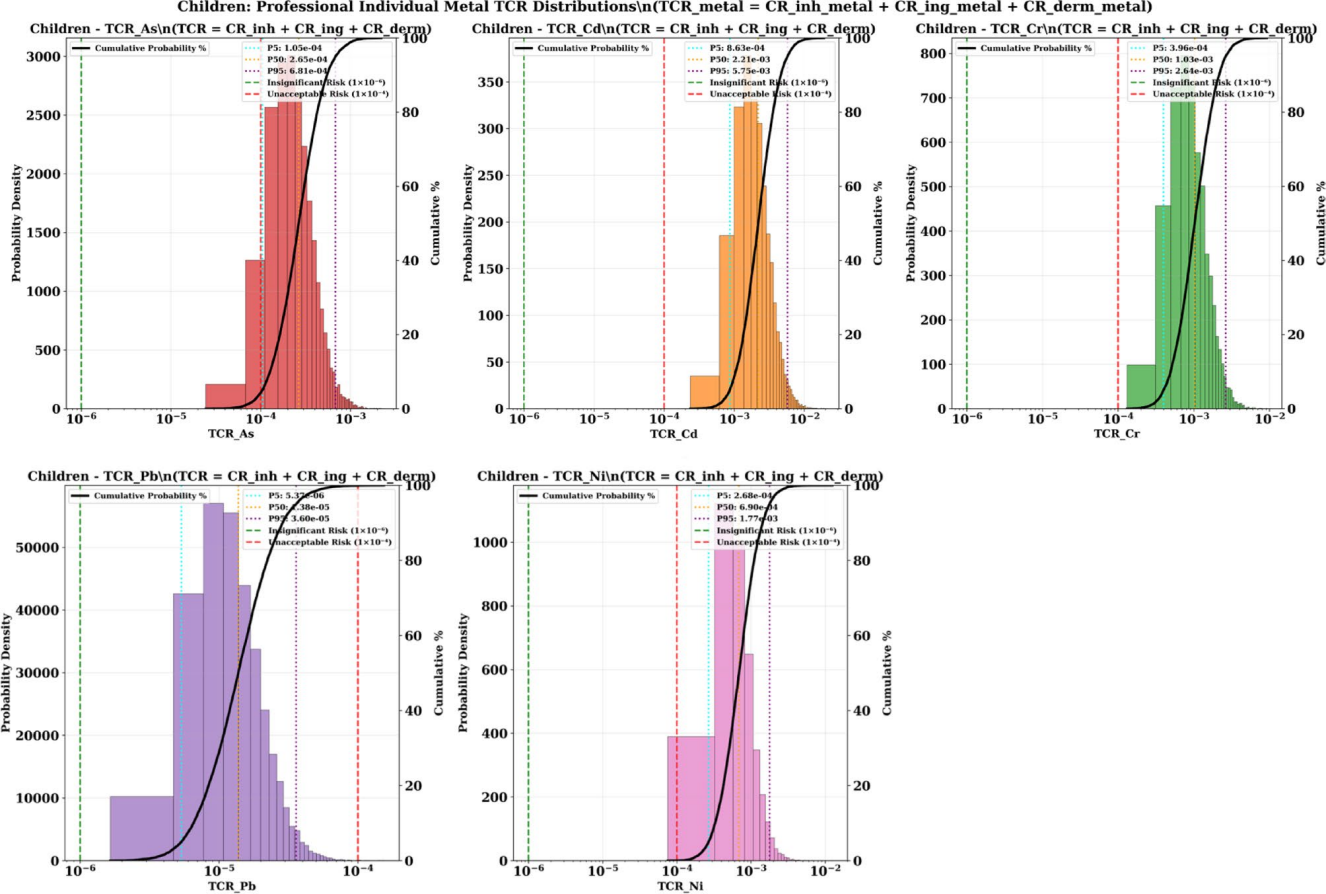

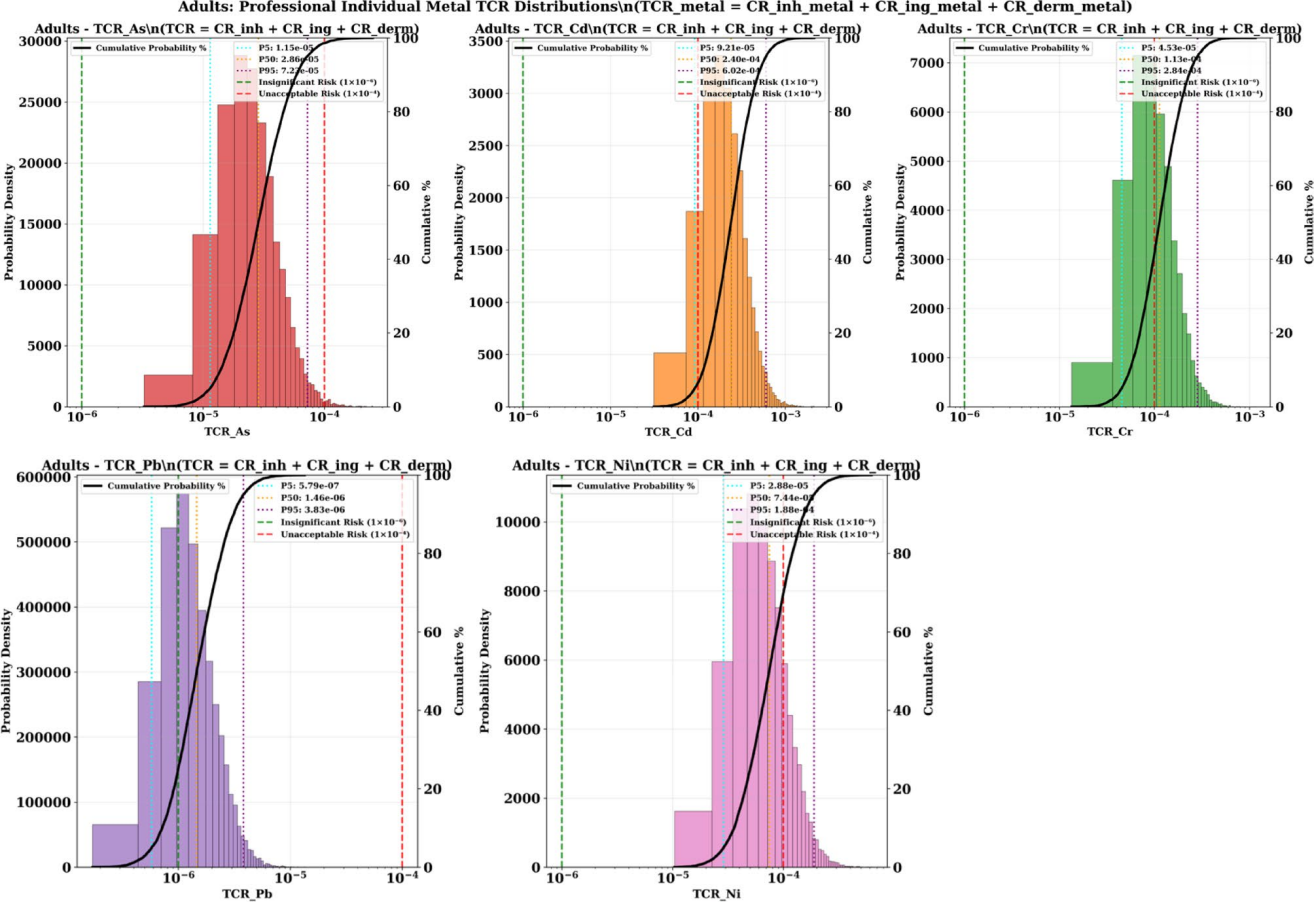
The results of cumulative probability % of TCR value in adult and children through different pathway exposure.

The pediatric population faces extreme carcinogenic health threats with multiple metals showing TCR values orders of magnitude above acceptable levels. Cadmium presents the most severe cancer emergency, with median (P50) TCR values of 2.21 × 10^−3^, representing 22 times the unacceptable cancer risk threshold. The 95th percentile (P95) cadmium TCR reaches 5.75 × 10^−3^, indicating that 5% of children face cancer risks 57 times above acceptable levels. These values suggest that the probability of exceeding acceptable cancer risk levels exceeds 95% for children from cadmium exposure alone (Fig. 11).

Chromium demonstrates similarly alarming cancer risks, with P50 values of 1.03 × 10^−3^ (10 times above threshold) and P95 values of 2.64 × 10^−3^ (26 times above threshold). The probability distributions indicate that approximately 90–95% of children exceed acceptable cancer risk levels from chromium exposure. Nickel shows substantial cancer risks with P50 values of 6.90 × 10^−4^ (7 times above threshold) and P95 values of 1.77 × 10^−3^ (18 times above threshold).

Arsenic contributes significant additional cancer burden with P50 values of 2.65 × 10^−4^ (2.7 times above threshold) and P95 values of 6.81 × 10^−4^ (6.8 times above threshold), indicating that approximately 70–80% of children exceed acceptable cancer risk levels from arsenic. Even lead, typically considered less carcinogenic, shows concerning cancer risks with P95 values of 3.6 × 10^−5^, approaching the unacceptable threshold.

The simultaneous exceedance of cancer risk thresholds for multiple carcinogenic metals creates an unprecedented cumulative cancer burden. Children face high probability of unacceptable cancer risks from at least one metal, with most children experiencing unacceptable risks from multiple metals simultaneously.

#### Adults

Adult cancer risks, while substantially lower than children’s, still represent serious public health concerns with multiple metals exceeding acceptable cancer risk thresholds. Cadmium remains the primary cancer threat for adults, with P50 values of 2.40 × 10^−4^ (2.4 times above threshold) and P95 values of 6.02 × 10^−4^ (6 times above threshold). These values indicate that approximately 70–80% of adults exceed acceptable cancer risk levels from cadmium exposure (Fig. 11).

Chromium shows moderate but concerning adult cancer risks, with P95 values of 2.84 × 10^−4^ (2.8 times above threshold), suggesting that approximately 30–40% of adults exceed acceptable cancer risk levels. Nickel demonstrates similar risk patterns with P95 values of 1.88 × 10^−4^ (1.9 times above threshold). Arsenic shows P95 values approaching the unacceptable threshold (7.23 × 10^−5^), while lead remains below concerning levels for adults.

The comparative analysis reveals dramatic age-related differences in cancer risks, with children showing 8–10 times higher TCR values than adults for most carcinogenic metals. Cadmium demonstrates the most extreme age difference, with children’s P50 values (2.21 × 10^−3^) being approximately 9 times higher than adults’ P50 values (2.40 × 10^−4^). Similar patterns emerge for chromium (9 × difference), nickel (9 × difference), and arsenic (9 × difference).

These quantitative differences confirm that physiological vulnerability factors create profound age-specific cancer risk patterns. However, unlike non-carcinogenic risks where adults achieved complete protection, the cancer risk analysis demonstrates that adults remain vulnerable to carcinogenic effects, particularly from cadmium exposure.

The Monte Carlo analysis provides unequivocal statistical evidence of a carcinogenic health emergency affecting the entire community. For children, the probability of exceeding acceptable cancer risk levels approaches 100% for cadmium, > 90% for chromium, > 80% for nickel, and > 70% for arsenic. These probabilities represent high probability of cancer risk exceedance across multiple carcinogenic metals.

For adults, the > 70% probability of cadmium cancer risk exceedance and > 30% probability for chromium indicate that the majority of the adult population also faces unacceptable cancer risks. The community-wide nature of the cancer emergency necessitates immediate comprehensive intervention strategies protecting all age groups.

The high probability provided by the Monte Carlo approach eliminates uncertainty about the need for emergency response and provides compelling scientific justification for immediate comprehensive intervention measures to protect the entire Menzel Bourguiba community from this unprecedented cancer threat.

### Risk management implications and validation

The sensitivity analysis results provide strategic guidance for risk management prioritization. The dominance of ingestion rate in determining risk levels suggests that interventions targeting soil ingestion behavior could achieve substantial risk reduction. Potential strategies include:

Behavioral interventions: Education programs promoting hand hygiene, reducing soil-to-mouth contact, and modifying outdoor activity patterns.

Environmental controls: Dust suppression measures, surface treatments to reduce soil availability, and creation of alternative play areas.

Institutional measures: Restrictions on certain outdoor activities in highly contaminated areas.

The high sensitivity to body weight reinforces the critical importance of age-specific protection strategies, with particular focus on young children who face the highest risks due to their lower body weights and higher exposure rates.

The moderate sensitivity to metal concentrations indicates that while environmental remediation remains important for long-term risk reduction, behavioral and institutional interventions may provide more immediate and cost-effective risk reduction in the short term.

The sensitivity analysis validates the risk assessment model structure by confirming that the parameters identified as most important (ingestion rate, body weight) align with established understanding of environmental exposure pathways and physiological vulnerability factors. The dominance of the ingestion pathway, as indicated by the high sensitivity to ingestion rate, is consistent with the Monte Carlo simulation results and deterministic risk assessment findings. The relatively low sensitivity to exposure duration, frequency, and other temporal parameters suggests that the chronic exposure assumptions used in the risk assessment are appropriate and that uncertainty in these parameters does not significantly affect risk conclusions. This finding strengthens confidence in the risk assessment approach and conclusions.

### Comparative analysis and literature context

The health risk levels documented in this study represent some of the most severe environmental health risks reported in the scientific literature. The combination of non-carcinogenic risks exceeding safety thresholds and cancer risks exceeding acceptable levels by orders of magnitude creates an unprecedented health threat profile. Comparison with other industrial contamination studies reveals the exceptional severity of the Menzel Bourguiba situation. While elevated cancer risks from heavy metal exposure have been reported around other industrial facilities^54,55^, the magnitude of excess cancer risk observed in this study (10–40 times acceptable levels) far exceeds typical findings. Most environmental cancer risk assessments report exceedances of 2–5 times acceptable levels, making the current findings particularly alarming. The simultaneous occurrence of non-carcinogenic and carcinogenic health risks affecting different age groups creates a complex public health challenge requiring comprehensive intervention strategies. The high probability provided by the Monte Carlo analysis eliminates uncertainty about the need for immediate action and provides robust scientific justification for emergency response measures.

## Multivariate statistical analysis

### Hierarchical cluster analysis (HCA)

**Fig. 12:**
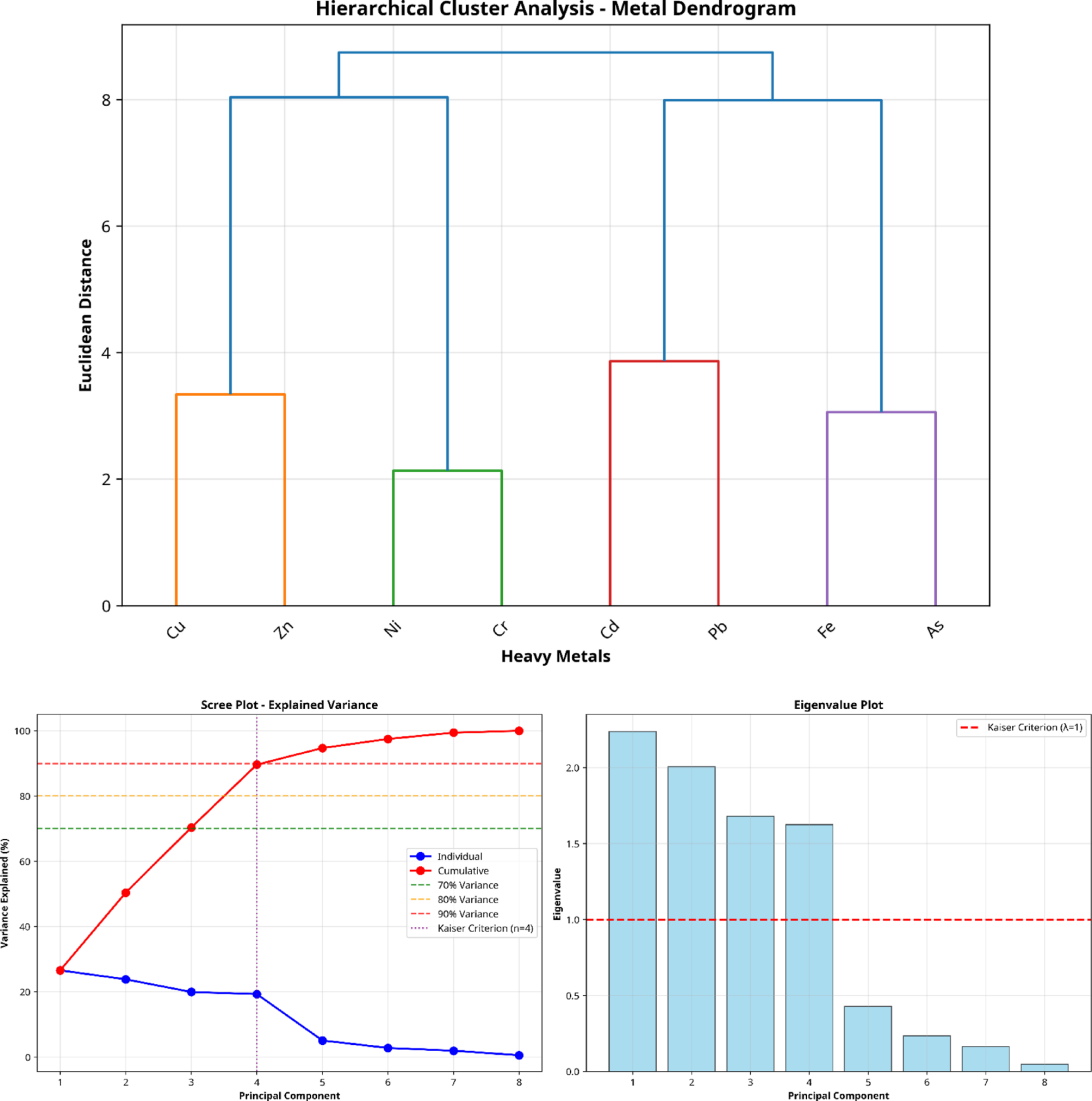

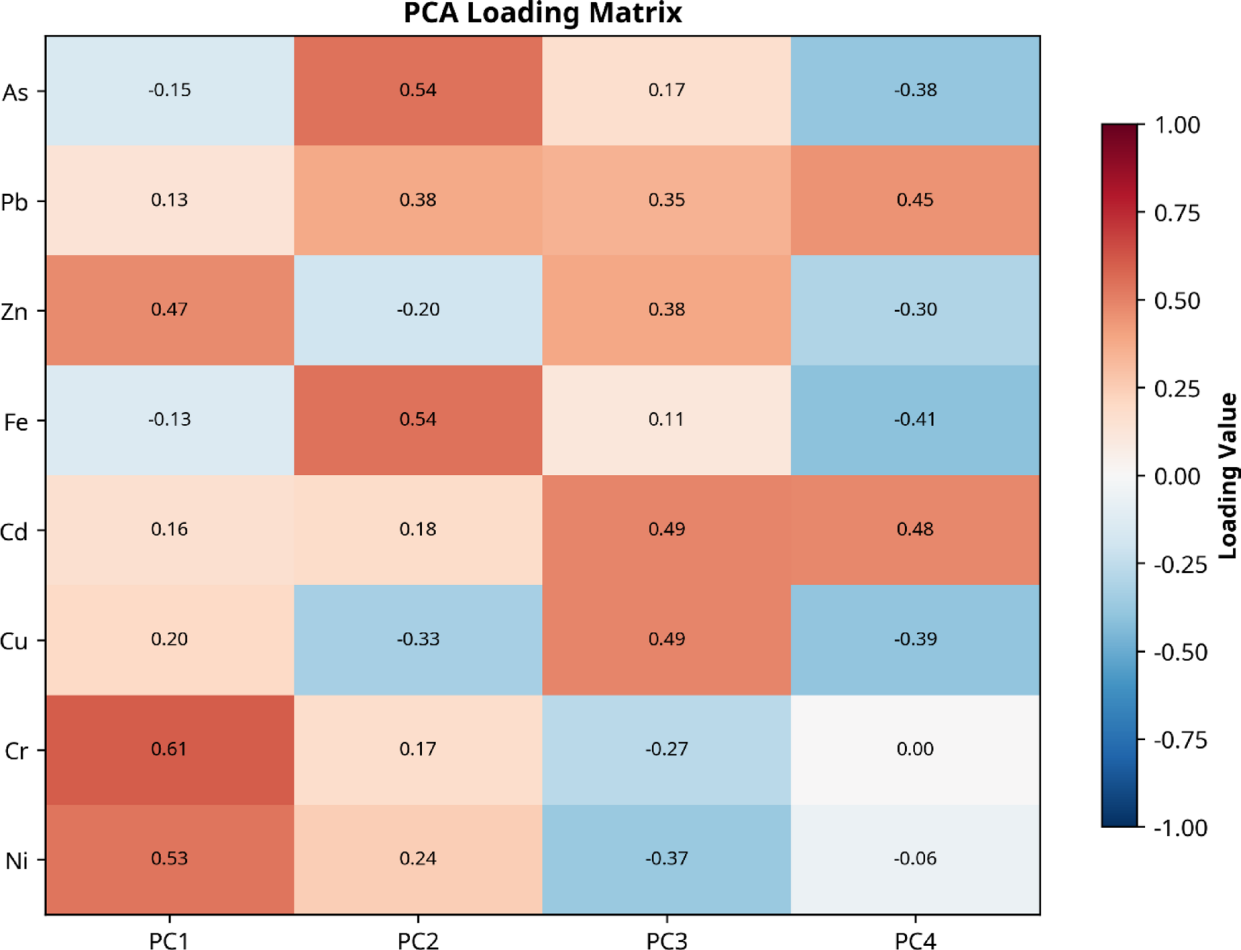
Cluster dendrogram of HMs in soil samples in the vicinity of Menzel Bourguiba steel smelter and PCA with optimum number of components (4 PCs) based on scree plot and variance.

The HCA (Fig. 12) with Ward’s linkage method and Euclidean distance metal clusters which showed particular geochemical relationships and pollution origin indicators. The two metals in Cluster 1 separated eight heavy metals in agricultural soil near the Menzel Bourguiba steel smelter into four distinct showed a strong connection because they share similar behavior during high-temperature metallurgical processes and they both originate from steel smelting operations. The two metals Ni and Cr formed Cluster 2 because they naturally occur together in Mediterranean clay soils which contain ultramafic parent materials and share similar geochemical characteristics in the regional geological area. The toxic metals Cd and Pb formed Cluster 3 which represents direct steel smelter emission contamination because these metals appear together in industrial combustion processes and follow identical atmospheric transport and deposition routes through agricultural areas. The facility’s hydrometallurgical iron processing operations created a distinct contamination source which Cluster 4 represents through its combination of Fe and As. The four-cluster arrangement successfully separated natural geogenic elements (Cluster 2: Ni–Cr) from various anthropogenic pollution sources (Clusters 1, 3 and 4) which demonstrated the diverse contamination effects of sixty years of industrial activities in this Mediterranean farming region.

#### Principal component analysis interpretation

The four principal components (Fig. 12) extracted from the analysis explained 90% of total variance in heavy metal data and showed different pollution sources of contamination and geochemical processes affecting agricultural soils near the Menzel Bourguiba steel smelter. The first principal component (PC1) showed positive loadings of 0.61 for Cr and 0.53 for Ni which indicated the natural background levels of these metals in Mediterranean clay soils because of local geological weathering and the natural chromium–nickel bond in regional parent materials. The second principal component (PC2) showed strong relationships between As (0.54) and Fe (0.54) which indicated a specific contamination factor that originated from hydrometallurgical iron processing activities which released arsenical compounds and iron-rich emissions that accumulated in surrounding agricultural lands through long-term atmospheric transport and clay soil retention. The third principal component (PC3) contained high values of Cd (0.49) and Cu (0.49) which indicated an anthropogenic contamination source stemming from high-temperature metallurgical activities that produced these metals during steel smelting operations which then accumulated in agricultural soils since 1962. The fourth principal component (PC4) showed significant loadings for Cd (0.48) and Pb (0.45) which indicated a possible secondary human-made factor stemming from particular emission events or waste management activities or combustion activities at the steel smelter facility. The PCA results explained 90% of total variance by separating natural soil formation processes from multiple human-made pollution sources which validated industrial activities as the main cause of heavy metal distribution in agricultural areas.

It should be noted that several PCA loadings fall in the moderate range (0.45–0.55), which limits the strength of the source apportionment conclusions. The PCA results should therefore be interpreted as indicative of probable pollution sources rather than definitive source identification. The convergence of PCA results with HCA findings and the spatial distribution patterns provide corroborating evidence for the proposed source assignments, but independent source confirmation (e.g., isotopic analysis or receptor modeling) would be required for definitive attribution.

### Limitations and uncertainties

Despite the comprehensive nature of this assessment, several limitations and uncertainties should be acknowledged. The soil sampling was conducted at a single time point, potentially missing temporal variability in contamination levels. Seasonal variations in metal mobility and bioavailability could influence exposure estimates, though the chronic exposure assumptions used in the assessment partially account for this variability. The exposure parameter values were derived from literature sources and may not perfectly represent the specific behavioral patterns and physiological characteristics of the local population. However, the sensitivity analysis indicates that uncertainty in these parameters does not significantly affect the overall risk conclusions, and the Monte Carlo approach incorporates parameter uncertainty in the risk estimates. The risk assessment focused on soil exposure pathways and did not include potential exposure through contaminated food crops or water sources. Additional exposure routes could increase total health risks beyond the levels estimated in this study, suggesting that the current assessment may represent a conservative estimate of total health impacts. Cumulative risk assessment across multiple chemicals was limited to metals analyzed in this study and did not include potential exposure to organic contaminants or other industrial pollutants that may be present in the area. The presence of additional contaminants could create synergistic effects that increase health risks beyond the additive model used in this assessment.

The absence of field replicates at individual sampling locations precludes a formal quantification of within-site spatial variability and sampling uncertainty. Future studies should incorporate co-located field duplicates to enable a rigorous assessment of sampling reproducibility. Nevertheless, the triplicate analytical replication (SD ≤ 5%) and certified reference material validation (recovery 90–110%) ensure that analytical uncertainty is well-controlled, and the Monte Carlo simulation framework partially addresses parameter uncertainty through probabilistic characterization of input distributions. The exclusion of food-chain exposure represents a significant limitation of this assessment. Future studies should incorporate crop sampling and bioavailability testing to enable a comprehensive multi-pathway exposure assessment. While geostatistical interpolation (e.g., kriging) can provide useful spatial predictions, the limited number of sampling points (n = 20) and their large spatial separation do not allow for reliable variogram modeling.

## Conclusions

The holistic assessment of heavy metal (HM) contamination around the Menzel Bourguiba steel smelter reveals a critical intersection between industrial legacy and public health vulnerability. Beyond the raw concentrations, the synthesis of pollution indexes (Igeo, EF, CF, and PLI) confirms a landscape of systematic degradation, where the soil is no longer merely a growth medium but a significant vector for toxic exposure.

The geochemical evidence specifically the extreme enrichment of Cadmium and Lead points to a clear anthropogenic signature that overwhelms natural crustal backgrounds. The transition from moderate to high contamination across various indexes suggests that the smelter’s influence is not localized but has created a regional footprint of persistent pollutants. The extreme EF values for Cd, in particular, indicate that current industrial mitigation strategies are insufficient to contain volatile heavy metal dispersion.

The most striking implication of this study is the stark age-related disparity in health risk. While adults remain relatively shielded from non-carcinogenic threats, the environmental burden shifts disproportionately to the pediatric population. The high probability of Hazard Index (HI) exceedance for children represents a looming public health risk, where lead exposure alone poses an immediate threat to neurodevelopmental outcomes. Furthermore, the exceedance of carcinogenic thresholds for both cohorts driven primarily by cadmium, chromium, and arsenic suggests that the risk is not a potential threat but an existing reality for the Menzel Bourguiba community.

To mitigate these risks, a multi-tiered intervention strategy is required:

- Immediate Clinical Intervention: Routine blood-lead level (BLL) screening for children in the immediate vicinity of the smelter to identify and treat acute toxicity.
- Source Control: Implementation of advanced particulate scrubbing technologies at the Menzel Bourguiba smelter to halt the ongoing deposition of Cd and Pb.
- Land-Use Policy: Temporary suspension of food-crop cultivation in extremely enriched zones, pivoting instead to non-edible phytoremediation crops to stabilize soil HM content.
- Systemic Monitoring: Transition from one-off assessments to a longitudinal environmental surveillance program that integrates soil health with community health records.

Ultimately, these findings serve as a mandatory call for a transition toward green metallurgical processing in Tunisia, balancing industrial economic output with the fundamental right to a non-toxic environment.

## Supplementary Information

Below is the link to the electronic supplementary material.

Supplementary Material 1

## Data Availability

“The datasets utilized and/or analyzed during the current study are available upon request from the corresponding author.”
