# Supplementary material for: Comprehensive assessment of heavy metal(loid) contamination and health risks in agricultural soils near the Menzel Bourguiba steel smelter, Tunisia
Source: Sci Rep. 2026 Apr 2;16:15806. doi: 10.1038/s41598-026-45034-x (PMC13194968; doi:10.1038/s41598-026-45034-x)
Supplement: Supplementary file 1 — Supplementary Material 1 [file 41598_2026_45034_MOESM1_ESM.docx]

**Table S1: Classification of environmental risk indices (*I_geo_, CF*, and *PLI).***

| **Index type** | | **Value** | **Environmental risk grade** | **Reference** |
| --- | --- | --- | --- | --- |
| Single indices | *I*_geo_ | *I*_geo_ ≤ 0 | Practically uncontaminated | Muller, 1979 |
|  |  | 0 < *I*_geo_ ≤ 1 | Uncontaminated to moderately contaminated |  |
|  |  | 1 < *I*_geo_ ≤ 2 | Moderately contaminated |  |
|  |  | 2 < *I*_geo_ ≤ 3 | Moderately to heavily contaminated |  |
|  |  | 3 < *I*_geo_ ≤ 4 | Heavily contaminated |  |
|  |  | 4 < *I*_geo_ ≤ 5 | Heavily to extremely contaminated |  |
|  |  | *I*_geo_ > 5 | Extremely contaminated |  |
|  | *CF* | *CF* < 1 | Low contamination | Hakanson, 1980 |
|  |  | 1 ≤ *CF* < 3 | Moderate contamination |  |
|  |  | 3 ≤ *CF* < 6 | Considerable contamination |  |
|  |  | *CF* ≥ 6 | High contamination |  |
|  | *PLI* | *PLI* < 1 | Unpolluted | Tomlinson et al., 1980 |
|  |  | *PLI* = 1 | Baseline levels of pollutants |  |
|  |  | *PLI* > 1 | Polluted |  |
|  | *EF* | < 2 | Deficiency to minimal enrichment (natural variability) | Saha et al., 2024 |
|  |  | 2 ≤ EF < 5 | Moderate enrichment |  |
|  |  | 5 ≤ EF < 20 | Significant enrichment |  |
|  |  | 20 ≤ EF < 40 | Very high enrichment |  |
|  |  | EF ≥ 40 | Extremely high enrichment |  |
|  |  |  |  |  |

| **Table S2** Description of exposure parameters used for ADD calculations | | | | | |
| --- | --- | --- | --- | --- | --- |
| **Symbol (unit)** | | **Parameter** | **Value** | | **Reference** |
|  |  |  | **Children** | **Adults** |  |
| IR_Ing_ (mg/d) | | Ingestion rate | 200 | 100 | (USEPA, 1996, 2002) |
| ED (yr) | | Exposure duration | 6 | 24 | (USEPA, 2002) |
| EF (d/yr) | | Exposure frequency | 350 | | (USEPA, 1996) |
| BW (kg) | | Average body weight | 15 | 70 | (USEPA, 2002) |
| AT (d) |  | Averaging time | 365 × ED | | (Dat et al., 2021; Malakootian et al., 2021) |
| IR_Inh_ (m^3^/d) | | Inhalation rate | 7.63 | 20 | (Ferreira-Baptista & De Miguel, 2005; USEPA, 2002; Xu et al., 2013) |
| PEF(m^3^/kg) | | Particle emission factor | 1.36×10^9^ | | (USEPA, 1996, 2002, 2011) |
| SA (cm^2^) | | Exposed skin surface area | 2800 | 5700 | (Dat et al., 2021; USEPA, 2002) |
| AF (mg/cm^2^/d) | | Skin-soil adherence factor | 0.2 | 0.07 | (USEPA, 2002) |
| ABS(unit-less) | | Dermal absorption factor | 0.001 | | (USEPA, 2002) |
| CF (kg/mg) | | Conversion factor | 1$\times$10^-6^ | | (USEPA, 2002) |

**Table S3** Reference doses (RfD) and cancer slope factors (CSF) for toxic metals via different exposure pathways(Adimalla, 2020; Al-Shidi et al., 2021; Dat et al., 2021; Malakootian et al., 2021; Tan et al., 2018; USEPA, 2002, 2011, 2012)

| Cd | Cr | As | Pb | Cu | Zn | Ni | Fe |
| --- | --- | --- | --- | --- | --- | --- | --- |
| **Reference dose (RfD) (mg/kg/day)** | | | | | | | |
| Ingestion pathway | | | | | | | |
| 1.00E-03 | 3.00E-03 | 3.00E-04 | 3.50E-03 | 4.00E-02 | 3.00E-01 | 2.00E-02 | 7.00E-01 |
| Dermal pathway | | | | | | | |
| 1.00E-05 | 6.00E-05 | 1.23E-04 | 5.25E-04 | 1.20E-02 | 6.00E-02 | 5.40E-03 | 4.50E-02 |
| Inhalation pathway | | | | | | | |
| 1.00E-05 | 2.86E-05 | 3.00E-04 | 3.50E-03 | 4.00E-02 | 3.00E-01 | 9.00E-05 | 2.20E-04 |
| **Cancer slope factor (CSF) (mg/kg/day)** | | | | | | | |
| Ingestion pathway | | | | | | | |
| 3.80E+01 | 5.00E-01 | 1.5 | 8.50E-03 | - | - | 8.40E-01 | - |
| Dermal pathway | | | | | | | |
| 3.80E+01 | 2.00E+00 | 3.66 | 5.30E-04 | - | - | 8.40E-01 | - |
| Inhalation pathway | | | | | | | |
| 6.30E+00 | 4.20E+01 | 15.1 | 8.50E-03 | - | - | 8.40E-01 | - |

**References**

Adimalla, N. (2020). Heavy metals contamination in urban surface soils of Medak province, India, and its risk assessment and spatial distribution. *Environmental Geochemistry and Health*, *42*(1), 59–75. https://doi.org/10.1007/s10653-019-00270-1

Al-Shidi, H. K., Sulaiman, H., Al-Reasi, H. A., Jamil, F., & Aslam, M. (2021). Human and ecological risk assessment of heavy metals in different particle sizes of road dust in Muscat, Oman. *Environmental Science and Pollution Research*, *28*(26), 33980–33993. https://doi.org/10.1007/s11356-020-09319-6

Dat, N. D., Nguyen, V.-T., Vo, T.-D.-H., Bui, X.-T., Bui, M.-H., Nguyen, L. S. P., Nguyen, X.-C., Tran, A. T.-K., Nguyen, T.-T.-A., Ju, Y.-R., Huynh, T.-M.-T., Nguyen, D.-H., Bui, H.-N., & Lin, C. (2021). Contamination, source attribution, and potential health risks of heavy metals in street dust of a metropolitan area in Southern Vietnam. *Environmental Science and Pollution Research*, *28*(36), 50405–50419. https://doi.org/10.1007/s11356-021-14246-1

Ferreira-Baptista, L., & De Miguel, E. (2005). Geochemistry and risk assessment of street dust in Luanda, Angola: A tropical

Hakanson, L. (1980). An ecological risk index for aquatic pollution control.a sedimentological approach. *Water Research*, *14*(8), 975–1001. https://doi.org/10.1016/0043-1354(80)90143-8

Malakootian, M., Mohammadi, A., Nasiri, A., Asadi, A. M. S., Conti, G. O., & Faraji, M. (2021). Spatial distribution and correlations among elements in smaller than 75 μm street dust: Ecological and probabilistic health risk assessment. *Environmental Geochemistry and Health*, *43*(1), 567–583. https://doi.org/10.1007/s10653-020-00694-0

Muller, G. (1969). Index of geoaccumulation in sediments of the Rhine River. *Geojournal*, *2*, 108–118.

Tan, Z., Lu, S., Zhao, H., Kai, X., Jiaxian, P., Win, M. S., Yu, S., Yonemochi, S., & Wang, Q. (2018). Magnetic, geochemical characterization and health risk assessment of road dust in Xuanwei and Fuyuan, China. *Environmental Geochemistry and Health*, *40*(4), 1541–1555. https://doi.org/10.1007/s10653-018-0070-7

Tomlinson, D.L., Wilson, J.G., Harris, C.R., Jeffrey, D.W., 1980. Problems in the assessment of heavy-metal levels in estuaries and the formation of a pollution index. Helgol¨ander Meeresuntersuchungen 33 (1–4), 566–575. https://doi.org/10.1007/ BF02414780. USEPA. (1996). *Soil Screening Guidance: User’s Guide*. U.S. Environmental Protection Agency: Office of Emergency and Remedial Response.

Saha A, Sen Gupta B, Patidar S,Martínez-Villegas N. (2024). Contamination risk assessment and distribution of rare trace metal(loid)s in surface soil of Cerrito Blanco, Mexico using various contamination indices. *Total Environ. Adv.9*, 200086.

USEPA. (2002). *SUPPLEMENTAL GUIDANCE FOR DEVELOPING SOIL SCREENING LEVELS FOR SUPERFUND SITES*. U.S. Environmental Protection Agency: Office of Emergency and Remedial Response.

USEPA. (2011). *Exposure Factors Handbook*. U.S. Environmental Protection Agency.

USEPA. (2012). *EPA’s Integrated Risk Information System Program*. U.S. Environmental Protection Agency: Office of Research and Development.

Xu, S., Zheng, N., Liu, J., Wang, Y., & Chang, S. (2013). Geochemistry and health risk assessment of arsenic exposure to street dust in the zinc smelting district, Northeast China. *Environmental Geochemistry and Health*, *35*(1), 89–99. https://doi.org/10.1007/s10653-012-9463-1

Yesilkanat, C. M., & Kobya, Y. (2021). Spatial characteristics of ecological and health risks of toxic heavy metal pollution from road d
